# Therapy’s Shadow: A Short History of the Study of Resistance to Cancer Chemotherapy

**DOI:** 10.3389/fphar.2013.00058

**Published:** 2013-05-07

**Authors:** Peter Keating, Alberto Cambrosio, Nicole C. Nelson, Andrei Mogoutov, Jean-Philippe Cointet

**Affiliations:** ^1^Department of History, Université du Québec à MontréalMontreal, QC, Canada; ^2^Department of Social Studies of Medicine, McGill UniversityMontreal, QC, Canada; ^3^IFRIS (Institut Francilien Recherche Innovation Société), Université Paris-EstMarne-la-Vallée, France; ^4^INRA-SenS (Sciences en Société), Université Paris-EstMarne-la-Vallée, France; ^5^Institut des Systèmes Complexes – Paris Île-de-FranceParis, France

**Keywords:** history of oncology, scientometrics, therapeutic resistance, targeted therapies

## Abstract

This article traces the history of research on resistance to drug therapy in oncology using scientometric techniques and qualitative analysis. Using co-citation analysis, we generate maps to visualize subdomains in resistance research in two time periods, 1975–1990 and 1995–2010. These maps reveal two historical trends in resistance research: first, a shift in focus from generic mechanisms of resistance to chemotherapy to a focus on resistance to targeted therapies and molecular mechanisms of oncogenesis; and second, a movement away from an almost exclusive reliance on animal and cell models and toward the generation of knowledge about resistance through clinical trial work. A close reading of highly cited articles within each subdomain cluster reveals specific points of transition from one regime to the other, in particular the failure of several promising theories of resistance to be translated into clinical insights and the emergence of interest in resistance to a new generation of targeted agents such as imatinib and trastuzumab. We argue that the study of resistance in the oncology field has thus become more integrated with research into cancer therapy – rather than constituting it as a separate domain of study, as it has done in the past, contemporary research treats resistance as the flip side to treatment, as therapy’s shadow.

## Introduction

Resistance to chemotherapy and other oncology drugs has long been a topic of interest to both researchers and clinicians, but how has research in this field changed over time, particularly with the introduction of targeted therapies for many common types of cancer? We argue that the study of resistance to cancer chemotherapy has undergone several major transformations over the last 15 years, both at the scientific and the organizational level. On the scientific side, the research field has seen a shift from generic mechanisms of resistance to chemotherapy to specific molecular mechanisms of cancer progression, corresponding with the advent of targeted cancer therapies. The discovery of oncogenes and oncogenic pathways has directed cancer therapeutics away from cell-kill to pathway disruption, and toward the creation of new drugs, mainly small molecules and antibodies. These new, targeted drugs do as much to unveil the biological processes of cancer initiation and progression as they do to shut them down.

Second, experimental work on resistance has moved from an almost exclusive focus on pre-clinical systems to clinical experimental studies. New forms of clinical research – biomarker trials, neo-adjuvant studies, and so on – have reoriented the experimental basis of resistance studies from animal models and cell lines to a mix of animal models, materials derived from human patients, and clinical studies. Rather than merely establishing drug efficacy, many clinical trials now function like a clinical experimental system, where the study of clinical efficacy is accompanied by a series of biological questions that treat resistance more as an integral component of therapy – therapy’s shadow, so to speak – and less as simply the failure of therapy.

## Mapping the Field

To analyze the development of the resistance field over time, we combined a qualitative review of the literature with a scientometric technique known as co-citation analysis (Small, 1973; Garfield et al., 1978; Vargas-Quesada and de Moya-Anegón, 2007). What is co-citation analysis and what are the advantages of this technique over simpler indicators, such as a list of most-cited articles? In contrast to bibliometric productivity measures (number of articles, number of citations given to articles, articles published in high impact-factor journals, etc.), co-citation mapping techniques use sophisticated information visualization tools that make complex relations and configurations visible without reducing them to a few, often crude statistical indicators. A list of articles is just that: a list of articles; co-citations networks provide not only insights about which articles researchers consider major contributions to the development of their domain, but they also enable us to visualize clusters of related references as the constitutive subdomains of a given research area at a given point in time.

Co-citation networks are one of two possible visualization techniques that can be used to map different aspects of a given field’s structure, development, and dynamics, the other, more easy-to-grasp, being inter-citation networks. Inter-citation is simply the association established when an article A (the citing reference) cites an article B (the cited reference). Co-citation is a subtler notion: two references A and B are co-cited if they frequently appear together in the reference list of other articles. Co-citation networks then display the most-cited references as nodes, with co-citation links connecting frequently co-cited references. The assumption is that cited references, because of their co-citation patterns, will be gathered together naturally into highly cohesive subgroups that provide a cognitive map of core contributions to a field, as they display the internal substructures and constitutive subdomains within that field. Inter-citation maps, in contrast, provide a more comprehensive view of a domain by showing the relations between a large number of references, even those that do not qualify as major contributions. Thus, inter-citation emphasizes the organizational components of a domain and co-citation its cognitive dynamics. In other words, whereas inter-citations capture the circulation of ideas throughout a given domain without hierarchical orderings, co-citations display the epistemic foundations of a given field at a particular point in time. An additional twist in the co-citation approach is to add a temporal axis to the resulting maps, in order to investigate the contribution of both older and more recent articles to the formation of a given domain. Rather than showing the actual historical development of a field, temporal co-citation maps reveal judgments about the relevant history of a field as perceived at a given moment by the authors of the articles used as source data.

Having opted, for the aforementioned reasons, to analyze the study of resistance using co-citations maps, we searched *Medline* for articles corresponding to the MeSH keyword (Drug Resistance, Neoplasms) introduced in 1995, and to the MeSH keywords (Drug Resistance AND Neoplasms) for the pre-1995 period. Resort to the MeSH ontology allowed us to capture not only articles that use the term “drug resistance” in their titles and abstract, but also, for instance, those that specifically focus on a drug while not explicitly mentioning that term. Since our goal was to analyze the transformation of the field, we selected articles corresponding to two different periods, the years 1976–1990 and 1995–2010, retrieving a set of 4,229 articles for the former and 18,822 articles for the latter. We positioned these two periods at the opposite ends of the time spectrum to better capture differences between early and recent research on resistance. The exact timespan of the two periods is somewhat arbitrary, as we could have chosen slightly shorter or longer periods, but this is inconsequential as co-citation analysis only retains the most-cited references and the most frequent co-citation links: both are relatively stable over short time periods. Finally, in order to test whether trends uncovered by the comparison of these two periods extended to the most recent years, we created a third dataset covering the years 2010 to November 2012.

While the MeSH keywords available in Medline allow for a very specific search strategy, a co-citation analysis requires lists of cited articles as provided by bibliometric databases such as Thomson Reuters’ *Web of Science* (*WoS*). Using a recently developed batch-matching technique to identify *WoS* references corresponding to *Medline* datasets (Leydesdorff and Opthof, 2013), we retrieved 16,162 matching references (86%) for the 1995–2010 period, and 3,145 matching references (74%) for the 1976–1990 period. The missing references belonged to journals not included in the *WoS*: while the coverage of biomedical journals is less extensive in *WoS* than PubMed, *WoS* included all most frequently cited journals. Finally, for the third dataset covering the years 2010–November 2012 we found 6,162 *Medline* references and 5,484 *WoS* matching references (89%).

We analyzed the three *WoS* datasets using the software platform *CorTexT* (http://manager.cortext.net/), which comprises algorithms designed to process bibliographic data and to perform several types of scientometric network analyses (Jones et al., 2011; Cointet et al., 2012). In the present case, we selected a distributional proximity measurement (Weeds and Weir, 2005) to calculate co-citation links between the 200 most-cited references. To display these links *CorTexT* applies a dynamic positioning algorithm that optimizes the location of all the nodes by minimizing the overall strain in the network. *CorTexT* also uses an automatic clustering algorithm to define (and color-code) co-citation clusters, i.e., cohesive subsets of the network that provide a high-level, fully bottom-up description of the network. To facilitate interpretation, *CorTexT* adds color circles around each cluster. Finally, we used *CorTexT’s* text-mining algorithms, based on a Natural Language Processing (NLP) techniques (Ananiadou and McNaught, 2006; Feldman and Sanger, 2007) to extract multi-term concepts from the titles and abstracts of the articles. Compared to the MeSH standardized keywords retrospectively added to references by indexers, NLP-based terms correspond to concepts actually used by the authors of articles. We were thus able to provide a preliminary, automatic description of a cluster’s content by characterizing each cluster with the concepts most specifically related to it. This was followed by a more detailed, manual inspection of individual references.

**Figure 1:**
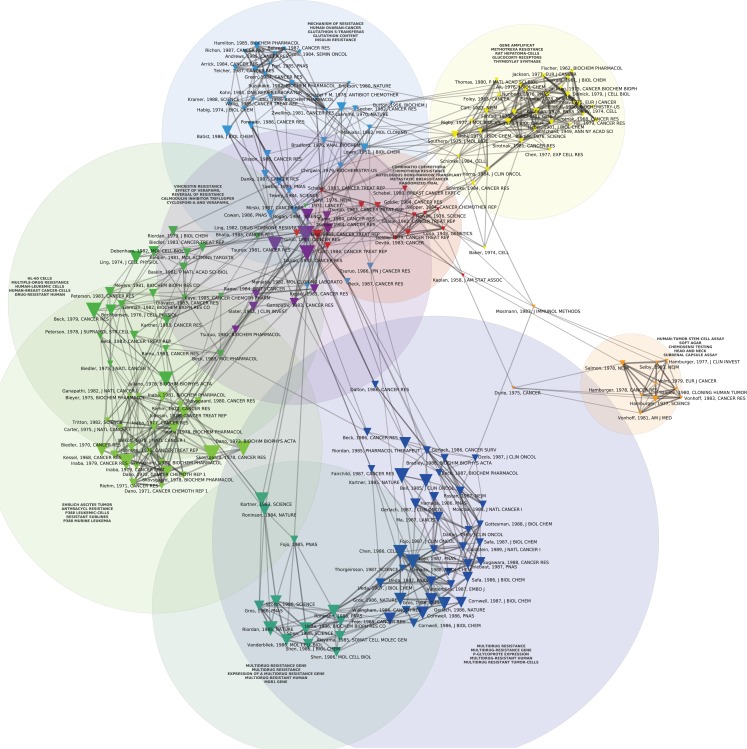
**Co-citation map of the 200 most-cited references in articles published from 1976 to 1990 in the domain of anti-neoplastic drug resistance**. The 200 most-cited references are represented by triangles whose size is proportional to the number of citations. Two references are connected in the co-citation network if they are frequently co-cited (i.e., jointly cited by other articles). Automatic clustering techniques are used to rearrange co-cited references into cohesive, color-coded sub-groups that provide a high-level description of the different thematic domains characterizing the field development. Natural-language processing algorithms are used to extract multi-term concepts from the titles and abstracts of the citing references, and the most specific multi-terms (as defined by a Chi-square measure) are used to tag each cluster, thus providing information about the thematic content of that cluster.

**Figure 2:**
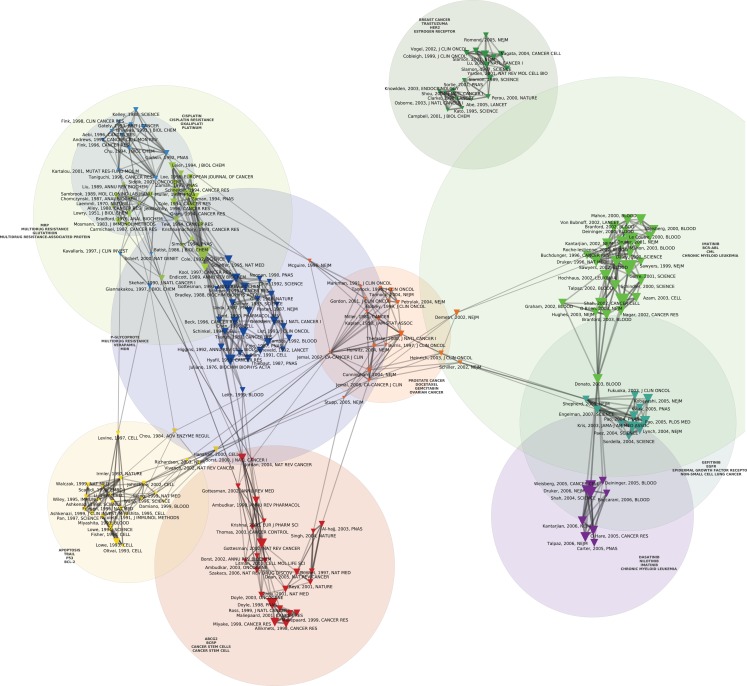
**Co Co-citation map of the 200 most-cited references in articles published from 1995 to 2010 in the domain of anti-neoplastic drug resistance**. See the legend of Figure 1 for explanations.

**Figure 3:**
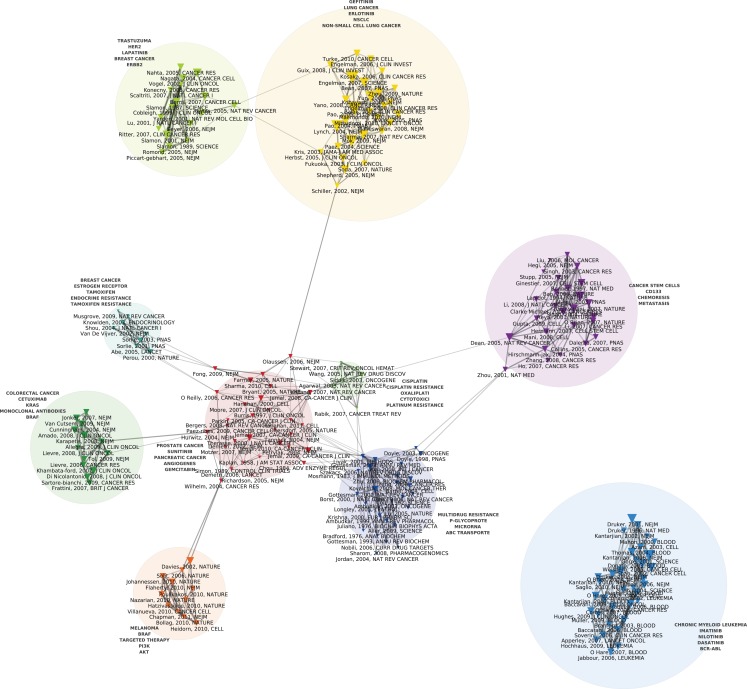
**Co-citation map of the 200 most-cited references in articles published from 2010 to 2012 in the domain of anti-neoplastic drug resistance**. See the legend of Figure 1 for explanations.

Figures 1 and 2 display the resulting maps for the 1976–1990 and 1995–2010 periods. A comparison of the two maps shows increased domain fragmentation. While each map displays distinct clusters, the map of the early period has a highly interconnected central component and the more recent period shows a number of specialized subdomains. Inspection of the clusters reveals that while the 1976–1990 map contains a number of centrally positioned, interconnected clusters related to the study of generic resistance mechanisms and multi-drug resistance (MDR), the 1995–2010 period is characterized by separate clusters that correspond to specific types of cancer and drugs directed against pathways closely connected to those individual pathologies. For example, we can identify clusters devoted to breast cancer, non-small cell lung cancer, prostate and ovarian cancer, and chronic myelogenous leukemia. In the case of chronic myelogenous leukemia, we have two distinct clusters that appear to correspond to different stages of development: first, to the initial exploration of the BCR-ABL translocation and the development of imatinib, followed by a second set of studies focusing on point mutations conferring resistance to imatinib, and the development of drugs designed to overcome this resistance. While MDR also appears as a cluster on the 1995–2010 map, the trend is clear: researchers’ attention has shifted from MDR to targeted therapies and related molecular mechanisms, in particular signaling pathways. In spite of recent calls to abandon histology (Bernards, 2012), this new perspective is still structured around the tissue of origin. The map of the 2010–2012 period (Figure 3), shows this trend even more clearly: in addition to a small MDR cluster (centered on P-glycoprotein and ABC-transporter: see below), a cluster on tumor stem cells and chemoresistance, and a cluster on platinum resistance, we see two distinct breast cancer clusters (one on endocrine resistance, hormone receptors, and tamoxifen, the other on HER2, trastzumab, and lapatinib), a colorectal cancer cluster (KRAS and cetuximab), a non-small cell lung cancer-gefitinib cluster, a melanoma cluster (BRAF and AKT), a chronic myelogenous leukemia cluster (imatinib, nilotinib, dasatinib), and a mixed prostate and pancreatic cancer cluster (sunitinib, angiogenesis, and gemcitabine).

In what follows, we outline several historical strands of resistance research in oncology and provide a more detailed, qualitative examination of the transition from one period to the other, based on the close reading of the key contributions displayed on the corresponding maps.

## Early Selection Models of Resistance and Their Clinical Correlates

**Figure 4:**
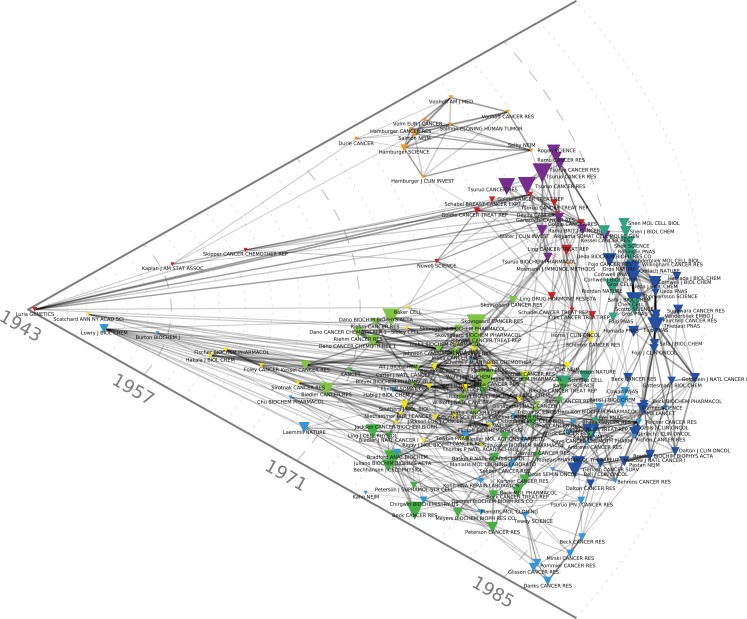
**Chronological co-citation map of the 200 most-cited references in articles published from 1976 to 1990 in the domain of anti-neoplastic drug resistance**. As compared to Figures 1–3, this Figure adds a logarithmic timeline and displays the components of each cluster chronologically.

The red cluster at the center of Figure 1 harbors the oldest reference of the map, namely the celebrated 1943 *Genetics* article by Luria and Delbrück (1943) on bacterial chemotherapy, which showed that it was not contact with antibiotics that produced resistance: resistance preceded therapy. In the particular case of bacterial resistance to viruses, resistant strains arose from mutations independently of contact with the virus. Resistance, in other words, was not an acquired immunity but the result of pre-existing heterogeneity that arose spontaneously in cultures developed from a single bacterium. The red cluster harbors another landmark paper, the 1964 article by Skipper et al. (1964) detailing the cell-kill hypothesis, according to which a successful cancer treatment entailed killing all cancer cells just as a successful antibiotic therapy entailed the elimination of all the pathogenic bacteria. Cancer chemotherapy in the 1960s was predicated on Skipper et al.’s (1964) hypothesis (aligned with the Luria and Delbrück findings) that unsuccessful therapy followed either the failure to eliminate all the bacteria/neoplastic cells or the emergence of resistant bacterial or cancerous clones. During this early period, mechanisms of resistance to cancer chemotherapies were thus modeled on bacterial chemotherapy and, in this regard, they were the mirror image of the putative mechanisms for chemotherapy’s success. Figure 4 allows us to better visualize these historical contributions. Based on the same dataset of Figure 1, using the same parameters and showing the same clusters, Figure 4 adds a (logarithmic) timeline thus ‘stretching’ each cluster to chronologically display its components. While other early papers (e.g., Scatchard, Löwry, Kaplan) correspond to methodological (statistical and biochemical) contributions that tend to have long citation careers, both the Luria and Delbrück, and the Skipper et al. (1964) paper refer to substantive, conceptual contributions that were clearly perceived as forerunners for subsequent work.

Another relatively early key contribution from the red cluster of Figure 1 that occupies a forerunner position in Figure 4 is Nowell’s (1976) article on the clonal evolution of tumor cell populations that is cited in recent work on tumor heterogeneity (Greaves, 2010) as the foundation of our current understanding of this phenomenon. Nowell’s contribution followed from a number of previous publications. They included Lloyd Law’s 1950s studies of the action of folic acid analogs in mouse leukemic cells. Law, in line with Luria and Delbrück hypothesis, found that “mutation and selection constitute the mechanism by which resistant leukemic cells develop” (Law, 1952). Parallel work by Makino (1956) in Japan transferred the bacterial growth hypothesis from the level of cells to the level of chromosomes, by developing an experimental system that produced consistent and characteristic chromosome patterns showing that the tumors developed from a single “stem cell.” Hauschka (1961), from the Roswell Park Cancer Institute, summarizing more than a decade’s work in this area in a number of animal and tissue systems, concluded that the initial cancer stem cell underwent a number of branching mutations in the course of tumor development that included not only secondary stem cell lines, but also chemotherapy and radiotherapy-resistant clones. The limits of the analysis were, however, well known at the time and, despite many advances, chromosome analysis and the novel somatic cell hybridization techniques were still considered blunt instruments unable to detect changes at the level of the gene. Hauschka’s complaint about his own techniques summarized a general frustration: “This furtiveness of the genes, coupled with the crudity of chromosomal hieroglyphs, irks the quantitatively minded investigator who wants to attack neoplasia in more articulate, molecular terms” (Hauschka, 1961). One of the reasons why these early publications, in contrast to Nowell’s (1976) contribution, did not make it to the top-200 references displayed on our maps, lies in the fact (mentioned by Nowell) that the genomic instability and heterogeneous nature of neoplasia had not been at the center of these initial studies. Further buttressed by immunological findings in the field of lymphoproliferative disorders, Nowell also drew a therapeutic implication for his model, absent in previous work, that has decidedly modern echoes. Given the heterogeneity of the clonal selection process, he suggested that “each patient’s cancer may require individual specific therapy and even this may be thwarted by emergence of a genetically variant subline resistant to the treatment” (Nowell, 1976).

Also harbored within the central red cluster of Figure 1 are three papers by Goldie et al. In the post-WWII period up to the end of the 1970s, much of the inquiry into the mechanisms of therapeutic resistance had been conducted outside of clinical research and, with the exception of Nowell’s musings about the future of cancer treatment, few lessons had been drawn from such models for therapy. The situation changed slightly, though not significantly, at the end of the 1970s with the appearance of the Goldie–Coldman hypothesis that proposed that tumor size could be related to the possibility that at least one resistant phenotype would emerge in the course of tumor development (Goldie and Coldman, 1979). For instance, the larger the tumor, the greater the possibility that it might harbor mutant clones. Drawing a direct line to therapy, Goldie and Coldman proposed that debulking could thus be seen as a means of reducing heterogeneity and thus the number of resistance clones. Alternating chemotherapies could also be rationalized as a means of eliminating resistant phenotypes (Goldie and Coldman, 1984). The *somatic mutation theory*, as they called it, could also be used to justify shortening the time between surgery and adjuvant therapy in order to decrease the possibility of the emergence of mutant, resistant clones.

Although they drew clinical implications from a generic model of resistance, in practice these propositions were justifications of ongoing activities, and had already been rationalized under the cell-kill hypothesis and its derivatives like the Norton–Simon hypothesis (Norton and Simon, 1977). The somatic mutation theory ultimately offered no specific guidelines for what to do in clinical research. As Goldie himself admitted: “While the somatic mutation theory provides predictions regarding broad strategic principles, it makes no statement about specific drugs, combinations, toxicity, etc. This sort of information will need to be derived from clinical trials, appropriate experimental systems, and the wisdom and experience of those who embark on these studies” (Goldie, 1983). In other words, while laboratory studies might offer a reason for clinical studies, they offered little direction. This would be a recurring theme in the field of studies of resistance for many years to come.

## Multi-Drug Resistance

The 1980s saw the consolidation of work on MDR, identified in shades of green and dark blue in the left and bottom of Figure 1. First isolated as instances of cross-resistance in animal cell lines selected for resistance to vinblastine and actinomycin D in the late 1960s and early 1970s (Kessel et al., 1968; Biedler and Riehm, 1970), by the second half of the 1970s, MDR was correlated with the expression of the P-glycoprotein (Juliano and Ling, 1976; Riordan and Ling, 1979). The discoverers of P-glycoprotein, J. R. Riordan’s group at the Hospital for Sick Children in Toronto, went on to clone the gene for the protein, *MDR-1* in 1985 (Riordan et al., 1985), the same year that the term “ATP-binding cassette” supergene family was defined (Gottesman et al., 2002).

At the NCI, the idea that MDR was the bottleneck in cancer chemotherapy became known as the “the gospel of Bruce Chabner” (the 1982–1995 Director of the NCI’s Division of Cancer Treatment). As noted previously, Figure 2 also includes a component devoted to MDR. As shown by the light green, dark blue, yellow, and red clusters that run from top to bottom of Figure 2, significant resources were directed to the study of MDR-1 throughout the 1980s (Endicott and Ling, 1989; Goldstein et al., 1989; Gottesman and Pastan, 1993; Gottesman and Ling, 2006). Interest in MDR mechanisms gained further interest with the discovery of the gene for a second generic drug pump, MRP, in 1992 (Cole et al., 1992) and the increasing entanglement of the biochemical mechanisms in oncogene and anti-oncogene (tumor suppressor genes) activities (Borst et al., 2000). Indeed, the year MRP was discovered, the promoter region of MDR-1 was found to be the target of RAS and p53 protein products. Mutant p53 genes, in particular, were found to activate rather than repress MDR-1 (Chin et al., 1992) Thereafter, p53 was found to regulate apoptosis and was thus implicated in a specific molecular pathway of resistance to anticancer agents in general (Lowe et al., 1993; Doyle et al., 1998). More recently, ABC drug transporters have crossed paths with models of cellular selection and the tumor stem cell theory (Dean et al., 2005). While ABC-transporters are held to protect tumor stem cells from chemotherapeutic agents thus creating resistant subpopulations that have the capacity for long-term renewal, this insight has yet to have a direct consequence for the clinic: “the stem-cell model of drug resistance at present has little applicability” (Dean et al., 2005).

Despite many successes in animals and cell culture that correspond in Figure 1 to the central purple cluster of highly co-cited articles that overlaps with the central red cluster (Tsuruo et al., 1981), the first clinical trial showing success in the use of a first-generation MDR inhibitor (cyclosporine) did not occur until 2001 (List et al., 2001). By then, more than 15 companies were pursuing research programs targeting novel, next-generation MDR-1 and MRP inhibitors seeking monoclonal antibodies, antisense oligonucleotides, and small molecules to block MDR activities (Persidis, 1999). The results have not been promising. As Tamaki et al. concluded a decade later: “Three generations of inhibitors later, we are still no closer to validating … the idea that increased chemotherapy efficacy can be achieved by inhibition of transporter-mediated efflux” (Tamaki et al., 2011; see also Robey et al., 2010; Falasca and Linton, 2012).

## Genetic Mechanisms of Resistance

The 1980s also saw the emergence of the first genetic mechanism for drug resistance: gene amplification of the dihydrofolate reductase (DHFR) genes in methotrexate resistance (yellow, top-right cluster of Figure 1). These genes had been implicated in mouse studies of methotrexate resistance since the mid-1970s (Flintoff et al., 1976) and by the early 1980s gene amplification in experimental animal systems had been described as a mechanism for resistance to methotrexate (Flintoff et al., 1982). By the mid 1980s, the mechanism had been generalized, at least hypothetically, to include all classes of anticancer drugs that affected the DNA of cancer cells. As one of the leaders of the field, the Stanford biologist Schimke (1984), put it in a review of recent studies: “We conclude that any number of agents that affect DNA synthesis or introduce damage into DNA may well facilitate amplification”.

And yet, like the results produced by MDR research, the gene amplification findings in methotrexate did not translate well into the clinic. Presenting a new human leukemia cell line to the scientific public, White et al. (1989) noted that previous experimental work on gene amplification had failed to predict clinical findings. In particular, they noted that: “Although amplification of DHFR genes in response to methotrexate exposure is well characterized experimentally, such gene amplification in human tumor tissue is not often reported in response to therapy and, when detected, is low in magnitude” (White et al., 1989). As illustrated by a statement in DeVita et al. (2005), according to which “despite many years of active investigation, the relative contribution of each of these different mechanisms to the development of cellular resistance to MTX remains unclear,” the situation remained unchanged well into the new century.

## The End of the Road and the Emergence of a New Regime

The absence of clinical correlations for the numerous experimental animal systems and *in vitro* human systems was a common problem that plagued the study of resistance. As the chapter on mechanisms of anti-neoplastic drug resistance in the fourth edition of DeVita et al.’s widely regarded *Cancer: Principles and Practice of Oncology* concluded: “Most of the mechanisms of drug resistance described have been determined in cell culture and animal models. … No comprehensive evaluation of mechanisms of resistance has been undertaken in human disease” (Morrow and Cowan, 1993). The following edition (Beck and Dalton, 1997) had little more to offer in terms of clinical findings but noted, however, that the mismatch between patients and animals in terms of thresholds of resistance made “a strong case” for “developing sensitive assays for markers of drug resistance and functional assays for the same to develop a ‘resistance profile’ of the individual patient’s tumor.” While clearly not a harbinger of personalized medicine, the proposal seemed to signal that traditional resistance studies had entered a *cul de sac*. Indeed, rather than rearranging the modalities of the traditional drug classes, the authors alluded to what would soon become known as targeted therapy, referring to the “developing knowledge of interactions of drug ‘target’ proteins” as a potent source of “radical change over the next few years” (Beck and Dalton, 1997). The fifth edition of the equally highly regarded *Holland-Frei Cancer Medicine* (2000) concluded along similar lines: despite clinical efforts to overcome resistance such as the “rational choice of conventional agents,” the future of resistance research lay in new agents (Morrow and Cowan, 2000).

And, indeed, Figure 2 displays a number of clusters that concern the new, targeted agents such as imatinib (followed by dasatinib, nilotinib), trastuzumab, and gefinitib. These targeted agents have been developed and tested in relation to specific kinds of cancer. On the right side of the map we see a green cluster displaying, in addition to the landmark paper by Daley et al. (1990) demonstrating that the expression of the bcr/abl protein could induce chronic myelogenous leukemia, a number of papers (Druker et al., 1996, 2001; Kantarjian et al., 2002; O’Brien et al., 2003) concerning one of the first and, so far, most successful targeted therapies, imatinib, deployed against chronic myelogenous leukemia and, later, gastrointestinal stromal tumor (a map, not shown, restricted to the 2006–2010 period shows a distinct gastrointestinal stromal tumor-imatinib cluster). As those involved in its development reported in 2002: “Based on the history of cancer therapeutics, the development of resistance to targeted agents, like imatinib could be expected. What was unusual, however, was the rapidity with which resistance mechanisms could be unraveled due to our thorough understanding of the drug action” (Mellinghoff and Sawyers, 2002). In the decade since those words were written, an even deeper knowledge of the drug mechanism has uncovered yet further mechanisms of resistance (Mahon et al., 2000; Gorre et al., 2001; Hochhaus et al., 2002; Shah et al., 2002; Vaidya et al., 2012) and drugs to combat that resistance (Shah et al., 2004): the purple cluster located at the bottom-right of the map denotes research on resistance to imatinib. The essential development, in this respect, is that resistance to targeted drugs, rather than an unqualified failure, is now seen as proof in principle that the drugs do indeed hit the target. In other words, “resistance conferring mutations can also be seen as the inevitable consequence of a drug-imposed selection process, which in fact confirms the validity of the targeted therapeutic approach” (Daub et al., 2004).

The other paradigmatic example of targeted therapy, trastuzumab, followed a similar path (Slamon et al., 2001). As we have shown in another paper (Cointet et al., 2012), breast cancer occupies a place of choice in the recent development of cancer genomics. On the top of the map we see another green cluster corresponding to work on resistance to trastuzumab. Although the precise mechanisms of resistance and action remain unknown 14 years after its FDA approval in 1998 (Stern, 2012) and 25 years after the initial correlation of HER2 amplification with survival and relapse (Slamon et al., 1987), multiple pathways, including a possible common node in the SRC pathway (Zhang et al., 2011), have been implicated. Indeed, notwithstanding the absence of a definitive mechanism, the study of the molecular pathways involved in trastuzumab resistance has led directly to therapeutic strategies to overcome resistance. This is the case, for example, with the Bolero series of clinical trials that target the PI3K/mTOR resistance pathway with everolimus (Tang and Finn, 2012). Similarly, resistance to BRAF inhibition in melanoma has cast light on the reactivation of the MAPK pathway as a mechanism of therapeutic failure thus opening the door for the clinical use of both BRAF inhibitors and complementary inhibitors of the MAPK pathway (Villanueva et al., 2011).

In the new world of targeted therapy, spectacular instances of resistance have continued to give rise to significant breakthroughs. For instance, in Figure 2 we see a cluster related to studies of samples from non-small cell lung cancer patients treated at the Massachusetts General Hospital with gefitinib. Those studies showed that although gefitinib produced dramatic improvement in only a small number of patients, those who did respond often had somatic mutations in the exons that encode for the TK domain of the EGFR (Lynch et al., 2004; Kobayashi et al., 2005). Researchers at Harvard and Dana Farber had published similar results the same year (2004) in *Science* (Paez et al., 2004; Pao et al., 2005). Analogous stories can be told in relation to the clusters displayed on Figure 3 that portrays the co-citation patterns of publications during the last 2 years. The trend is thus clear: whereas the pathways of disease progression and therapeutic resistance have in the past been considered separate, distinct processes, they are now treated as common biopathological pathways (Alexander and Friedl, 2012).

## Conclusion

As argued by Jones et al. (2011), new scientometric and text analysis techniques as applied to large datasets of publications provide an innovative way of mapping the structure of a scientific field and its development (Boyack et al., 2005; Börner, 2010). The maps we discussed in this paper document a transition from generic mechanisms of resistance to chemotherapy to a focus on resistance to targeted therapies and molecular mechanisms of ontogenesis, a finding that might resonate with many practitioners’ experiences of the field. Our demonstration, however, is based on the analysis of a comprehensive dataset of publications, the systematic resort to quantitative indicators derived from this dataset in combination with network analysis and NLP algorithms, and data visualization techniques. As such, it shows that such a transition is not just a subjective interpretation of the current state of clinical research in oncology, but can be tracked through the analysis of changing citation patterns in the literature. Moreover, our analysis includes a detailed description of connections and structural relationships between subdomains, thus providing insights into the differential topology of clusters of publications at different times: by comparing, for instance, Figure 1 with Figure 3, one can easily visualize differences in the degree of integration or fragmentation of subdomains at these two different periods.

Moreover, some of results we report provide an interesting take on recent debates within oncology, namely whether histological classifications of the anatomic origin of tumors are still relevant to cancer treatment in the post-genomic era, or whether treatments should be based solely on the molecular profile of the tumor without considering histology. Our analysis shows that while molecular biomarkers and the study of pathways do indeed make-up a large part of the recent literature, these developments take place within subdomains that are still best characterized by anatomically defined diseases. With these new lines of research into the molecular mechanisms of resistance and their relationship to targeted drugs, the contemporary study of drug resistance in oncology has succeeded in establishing a greater degree of clinical relevance than early selection models of resistance. Our analysis also demonstrates a concomitant shift in the methods used to study resistance, from an almost exclusive reliance on animal models to a focus on using human tissue samples and clinical trials as a site of generating new information about resistance. These findings point to the growing importance of clinical trials as a site for generating knowledge about basic biological questions such as the mechanisms of resistance, rather than merely as a site for testing the safety and efficacy of new drugs.

This historical shift has also effected organizational changes in the structure of the research field with potential implications for the future study of resistance. While contemporary trends show a merging of resistance research with clinical research and even treatment, they also show, as just noted, the increasing fragmentation of a domain that was once defined by a common interest in MDR. The trend toward the separation of resistance research activities into loosely connected disease and treatment-based clusters suggests an ongoing need for increased communication and collaboration between researchers working on the molecular mechanisms of drug resistance. In particular, it raises the thorny issue of the relation between the traditional division of the oncology domain into separate specialties defined by tissue of origin, and transversal research activities based on common molecular mechanisms in different kinds of cancer.

## Conflict of Interest Statement

The authors declare that the research was conducted in the absence of any commercial or financial relationships that could be construed as a potential conflict of interest.

## Supplementary Material

The Supplementary Material for this article can be found online at http://www.frontiersin.org/Pharmacology_of_Anti-Cancer_Drugs/10.3389/fphar.2013.00058/abstract
