# Supplementary figures and images for: Therapy’s Shadow: A Short History of the Study of Resistance to Cancer Chemotherapy
Source: Front Pharmacol. 2013 May 7;4:58. doi: 10.3389/fphar.2013.00058 (PMC3646244; doi:10.3389/fphar.2013.00058)

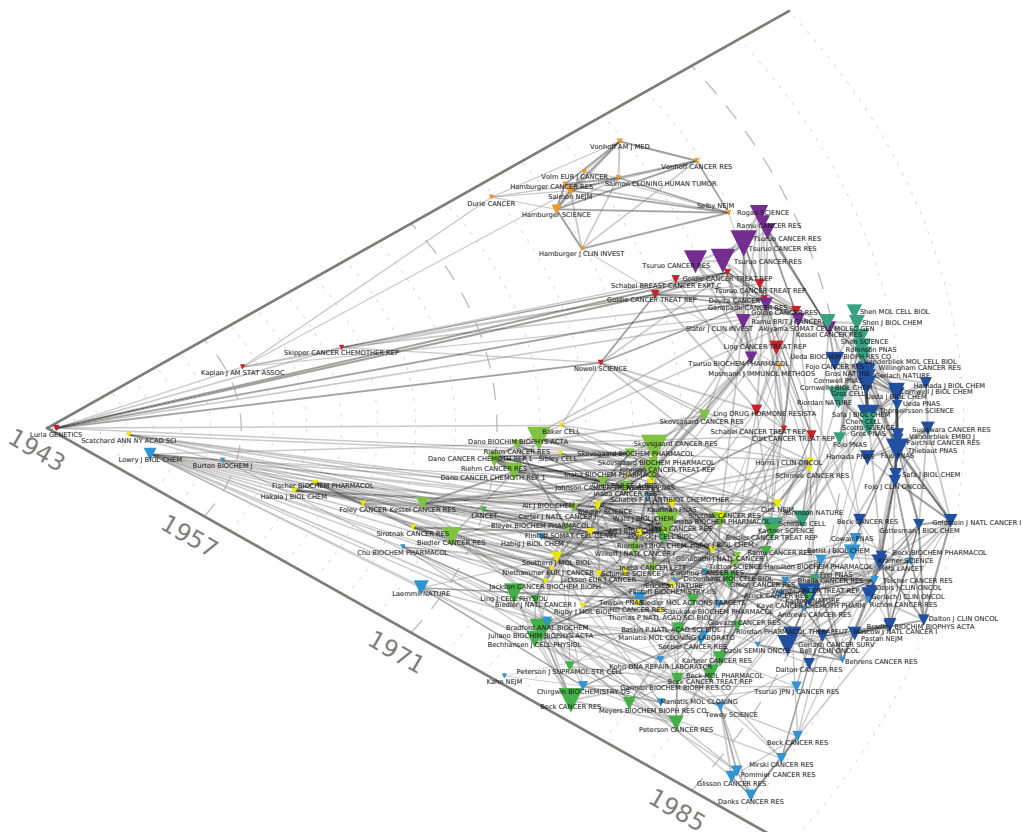

Supplement: Supplementary file 4 [file 42120_Keating_Presentation4.PDF]
