# Supplementary material for: Therapy’s Shadow: A Short History of the Study of Resistance to Cancer Chemotherapy
Source: Front Pharmacol. 2013 May 7;4:58. doi: 10.3389/fphar.2013.00058 (PMC3646244; doi:10.3389/fphar.2013.00058)
Supplement: Supplementary file 5 [file 42120_Keating_DataSheet1.PDF]

SUPPLEMENTAL MATERIAL

Top 200 cited references during the 1976-1990 period

Short reference (on maps)	Detailed reference	Nr. of citations
SKOVSGAARD T, 1978, CANCER RES	SKOVSGAARD T, 1978, CANCER RES, V38, P1785	252
TSURUO T, 1983, CANCER RES	TSURUO T, 1983, CANCER RES, V43, P2905	239
LOWRY OH, 1951, J BIOL CHEM	LOWRY OH, 1951, J BIOL CHEM, V193, P265	225
INABA M, 1979, CANCER RES	INABA M, 1979, CANCER RES, V39, P2200	218
TSURUO T, 1982, CANCER RES	TSURUO T, 1982, CANCER RES, V42, P4730	208
TSURUO T, 1981, CANCER RES	TSURUO T, 1981, CANCER RES, V41, P1967	186
DANO K, 1973, BIOCHIM BIOPHYS ACTA	DANO K, 1973, BIOCHIM BIOPHYS ACTA, V323, P466	178
BECK WT, 1979, CANCER RES	BECK WT, 1979, CANCER RES, V39, P2070	170
JULIANO RL, 1976, BIOCHIM BIOPHYS ACTA	JULIANO RL, 1976, BIOCHIM BIOPHYS ACTA, V455, P152	161
KARTNER N, 1985, NATURE	KARTNER N, 1985, NATURE, V316, P820	153
LAEMMLI UK, 1970, NATURE	LAEMMLI UK, 1970, NATURE, V227, P680	151
FOJO AT, 1987, P NATL ACAD SCI USA	FOJO AT, 1987, P NATL ACAD SCI USA, V84, P265	142
KARTNER N, 1983, SCIENCE	KARTNER N, 1983, SCIENCE, V221, P1285	135
RIORDAN JR, 1985, NATURE	RIORDAN JR, 1985, NATURE, V316, P817	128
JOHNSON RK, 1978, CANCER TREAT REP	JOHNSON RK, 1978, CANCER TREAT REP, V62, P1535	127
CHEN CJ, 1986, CELL	CHEN CJ, 1986, CELL, V47, P381	126
BIEDLER JL, 1970, CANCER RES	BIEDLER JL, 1970, CANCER RES, V30, P1174	119
LING V, 1974, J CELL PHYSIOL	LING V, 1974, J CELL PHYSIOL, V83, P103	119
DANO K, 1972, CANCER CHEMOTH REP 1	DANO K, 1972, CANCER CHEMOTH REP 1, V56, P701	117
INABA M, 1978, BIOCHEM PHARMACOL	INABA M, 1978, BIOCHEM PHARMACOL, V27, P2123	116
ROGAN AM, 1984, SCIENCE	ROGAN AM, 1984, SCIENCE, V224, P994	113
BELL DR, 1985, J CLIN ONCOL	BELL DR, 1985, J CLIN ONCOL, V3, P311	113
BRADFORD MM, 1976, ANAL BIOCHEM	BRADFORD MM, 1976, ANAL BIOCHEM, V72, P248	110
GERLACH JH, 1986, NATURE	GERLACH JH, 1986, NATURE, V324, P485	109
HAMBURGER AW, 1977, SCIENCE	HAMBURGER AW, 1977, SCIENCE, V197, P461	107
SALMON SE, 1978, NEW ENGL J MED	SALMON SE, 1978, NEW ENGL J MED, V298, P1321	104
BATIST G, 1986, J BIOL CHEM	BATIST G, 1986, J BIOL CHEM, V261, P5544	99
GOLDIE JH, 1979, CANCER TREAT REP	GOLDIE JH, 1979, CANCER TREAT REP, V63, P1727	98
RIORDAN JR, 1985, PHARMACOL THERAPEUT	RIORDAN JR, 1985, PHARMACOL THERAPEUT, V28, P51	97
GROS P, 1986, CELL	GROS P, 1986, CELL, V47, P371	96
SOUTHERN EM, 1975, J MOL BIOL	SOUTHERN EM, 1975, J MOL BIOL, V98, P503	95
GROS P, 1986, NATURE	GROS P, 1986, NATURE, V323, P728	92
FOJO A, 1985, CANCER RES	FOJO A, 1985, CANCER RES, V45, P3002	89
ALT FW, 1978, J BIOL CHEM	ALT FW, 1978, J BIOL CHEM, V253, P1357	87
CORNWELL MM, 1986, P NATL ACAD SCI USA	CORNWELL MM, 1986, P NATL ACAD SCI USA, V83, P3847	84
RONINSON IB, 1986, P NATL ACAD SCI USA	RONINSON IB, 1986, P NATL ACAD SCI USA, V83, P4538	83
PASTAN I, 1987, NEW ENGL J MED	PASTAN I, 1987, NEW ENGL J MED, V316, P1388	81
BECHHANSEN NT, 1976, J CELL PHYSIOL	BECHHANSEN NT, 1976, J CELL PHYSIOL, V88, P23	79
VANDERBLIEK AM, 1986, MOL CELL BIOL	VANDERBLIEK AM, 1986, MOL CELL BIOL, V6, P1671	77
SHEN DW, 1986, SCIENCE	SHEN DW, 1986, SCIENCE, V232, P643	77
HAMILTON TC, 1985, BIOCHEM PHARMACOL	HAMILTON TC, 1985, BIOCHEM PHARMACOL, V34, P2583	75

RONINSON IB, 1984, NATURE	RONINSON IB, 1984, NATURE, V309, P626	74
GOLDIE JH, 1984, CANCER RES	GOLDIE JH, 1984, CANCER RES, V44, P3643	72
GOLDSTEIN LJ, 1989, J NATL CANCER I	GOLDSTEIN LJ, 1989, J NATL CANCER I, V81, P116	71
TSURUO T, 1984, CANCER RES	TSURUO T, 1984, CANCER RES, V44, P4303	71
SCOTTO KW, 1986, SCIENCE	SCOTTO KW, 1986, SCIENCE, V232, P751	70
MANIATIS T, 1982, MOL CLONING LABORATO	MANIATIS T, 1982, MOL CLONING LABORATO	69
CORNWELL MM, 1987, J BIOL CHEM	CORNWELL MM, 1987, J BIOL CHEM, V262, P2166	69
HAMADA H, 1986, P NATL ACAD SCI USA	HAMADA H, 1986, P NATL ACAD SCI USA, V83, P7785	68
CURT GA, 1984, CANCER TREAT REP	CURT GA, 1984, CANCER TREAT REP, V68, P87	67
RIORDAN JR, 1979, J BIOL CHEM	RIORDAN JR, 1979, J BIOL CHEM, V254, P2701	67
MA DDF, 1987, LANCET	MA DDF, 1987, LANCET, V1, P135	67
TOWBIN H, 1979, P NATL ACAD SCI USA	TOWBIN H, 1979, P NATL ACAD SCI USA, V76, P4350	67
LING V, 1983, CANCER TREAT REP	LING V, 1983, CANCER TREAT REP, V67, P869	67
GERLACH JH, 1987, J CLIN ONCOL	GERLACH JH, 1987, J CLIN ONCOL, V5, P1452	66
THIEBAUT F, 1987, P NATL ACAD SCI USA	THIEBAUT F, 1987, P NATL ACAD SCI USA, V84, P7735	65
KARTNER N, 1983, CANCER RES	KARTNER N, 1983, CANCER RES, V43, P4413	65
DEBENHAM PG, 1982, MOL CELL BIOL	DEBENHAM PG, 1982, MOL CELL BIOL, V2, P881	64
MOSCOW JA, 1988, J NATL CANCER I	MOSCOW JA, 1988, J NATL CANCER I, V80, P14	64
Luria SE, 1943, GENETICS	Luria SE, 1943, GENETICS, V28, P491	63
GOLDIE JH, 1982, CANCER TREAT REP	GOLDIE JH, 1982, CANCER TREAT REP, V66, P439	63
KESSEL D, 1985, CANCER RES	KESSEL D, 1985, CANCER RES, V45, P1687	63
SLATER LM, 1982, J CLIN INVEST	SLATER LM, 1982, J CLIN INVEST, V70, P1131	63
AKIYAMA SI, 1985, SOMAT CELL MOLEC GEN	AKIYAMA SI, 1985, SOMAT CELL MOLEC GEN, V11, P117	63
BRADLEY G, 1988, BIOCHIM BIOPHYS ACTA	BRADLEY G, 1988, BIOCHIM BIOPHYS ACTA, V948, P87	62
GLISSON B, 1986, CANCER RES	GLISSON B, 1986, CANCER RES, V46, P1934	62
PETERSON RHF, 1983, CANCER RES	PETERSON RHF, 1983, CANCER RES, V43, P222	62
KESSEL D, 1968, CANCER RES	KESSEL D, 1968, CANCER RES, V28, P938	61
KAUFMAN RJ, 1979, P NATL ACAD SCI USA	KAUFMAN RJ, 1979, P NATL ACAD SCI USA, V76, P5669	61
RIEHM H, 1971, CANCER RES	RIEHM H, 1971, CANCER RES, V31, P409	60
RAMU A, 1984, CANCER RES	RAMU A, 1984, CANCER RES, V44, P144	60
POMMIER Y, 1986, CANCER RES	POMMIER Y, 1986, CANCER RES, V46, P3075	59
SAFA AR, 1986, J BIOL CHEM	SAFA AR, 1986, J BIOL CHEM, V261, P6137	59
UEDA K, 1987, P NATL ACAD SCI USA	UEDA K, 1987, P NATL ACAD SCI USA, V84, P3004	58
INABA M, 1977, CANCER RES	INABA M, 1977, CANCER RES, V37, P4629	57
INABA M, 1981, BIOCHEM PHARMACOL	INABA M, 1981, BIOCHEM PHARMACOL, V30, P1863	56
SCATCHARD G, 1949, ANN NY ACAD SCI	SCATCHARD G, 1949, ANN NY ACAD SCI, V51, P660	56
BECK WT, 1983, CANCER TREAT REP	BECK WT, 1983, CANCER TREAT REP, V67, P875	55
GERLACH JH, 1986, CANCER SURV	GERLACH JH, 1986, CANCER SURV, V5, P25	55
GROS P, 1986, P NATL ACAD SCI USA	GROS P, 1986, P NATL ACAD SCI USA, V83, P337	55
WILKOFF LJ, 1978, J NATL CANCER I	WILKOFF LJ, 1978, J NATL CANCER I, V61, P1521	55
CHIRGWIN JM, 1979, BIOCHEMISTRY-US	CHIRGWIN JM, 1979, BIOCHEMISTRY-US, V18, P5294	54
TEICHER BA, 1987, CANCER RES	TEICHER BA, 1987, CANCER RES, V47, P388	54
SHEN DW, 1986, J BIOL CHEM	SHEN DW, 1986, J BIOL CHEM, V261, P7762	54
GANAPATHI R, 1983, CANCER RES	GANAPATHI R, 1983, CANCER RES, V43, P3696	54

BIEDLER JL, 1983, CANCER TREAT REP	BIEDLER JL, 1983, CANCER TREAT REP, V67, P859	53
KOHN KW, 1981, DNA REPAIR LABORATOR	KOHN KW, 1981, DNA REPAIR LABORATOR, P379	52
SUZUKAKE K, 1982, BIOCHEM PHARMACOL	SUZUKAKE K, 1982, BIOCHEM PHARMACOL, V31, P121	51
DALTON WS, 1989, J CLIN ONCOL	DALTON WS, 1989, J CLIN ONCOL, V7, P415	51
BIEDLER JL, 1981, MOL ACTIONS TARGETS	BIEDLER JL, 1981, MOL ACTIONS TARGETS, P453	50
JOHNSON RK, 1976, CANCER TREAT REP	JOHNSON RK, 1976, CANCER TREAT REP, V60, P99	50
TRITTON TR, 1982, SCIENCE	TRITTON TR, 1982, SCIENCE, V217, P248	50
BECK WT, 1987, BIOCHEM PHARMACOL	BECK WT, 1987, BIOCHEM PHARMACOL, V36, P2879	49
SIBLEY CH, 1974, CELL	SIBLEY CH, 1974, CELL, V2, P221	49
NUNBERG JH, 1978, P NATL ACAD SCI USA	NUNBERG JH, 1978, P NATL ACAD SCI USA, V75, P5553	49
SCHABEL FM, 1980, BREAST CANCER EXPT C	SCHABEL FM, 1980, BREAST CANCER EXPT C, P199	49
COWAN KH, 1986, P NATL ACAD SCI USA	COWAN KH, 1986, P NATL ACAD SCI USA, V83, P9328	49
BASKIN F, 1981, P NATL ACAD SCI-BIOL	BASKIN F, 1981, P NATL ACAD SCI-BIOL, V78, P3654	48
UEDA K, 1986, BIOCHEM BIOPH RES CO	UEDA K, 1986, BIOCHEM BIOPH RES CO, V141, P956	47
TSURUO T, 1982, BIOCHEM PHARMACOL	TSURUO T, 1982, BIOCHEM PHARMACOL, V31, P3138	47
LING V, 1982, DRUG HORMONE RESISTA	LING V, 1982, DRUG HORMONE RESISTA, V1, P1	47
VONHOFF DD, 1981, AM J MED	VONHOFF DD, 1981, AM J MED, V70, P1027	46
GOTTESMAN MM, 1988, J BIOL CHEM	GOTTESMAN MM, 1988, J BIOL CHEM, V263, P12163	46
SUGAWARA I, 1988, CANCER RES	SUGAWARA I, 1988, CANCER RES, V48, P1926	46
FLINTOFF WF, 1976, SOMAT CELL GENET	FLINTOFF WF, 1976, SOMAT CELL GENET, V2, P245	45
CARTER SK, 1975, J NATL CANCER I	CARTER SK, 1975, J NATL CANCER I, V55, P1265	45
CORNWELL MM, 1986, J BIOL CHEM	CORNWELL MM, 1986, J BIOL CHEM, V261, P7921	45
SHEN DW, 1986, MOL CELL BIOL	SHEN DW, 1986, MOL CELL BIOL, V6, P4039	45
SKOVSGAARD T, 1977, BIOCHEM PHARMACOL	SKOVSGAARD T, 1977, BIOCHEM PHARMACOL, V26, P215	45
GARMAN D, 1982, BIOCHEM BIOPH RES CO	GARMAN D, 1982, BIOCHEM BIOPH RES CO, V105, P157	45
TSURUO T, 1986, JPN J CANCER RES	TSURUO T, 1986, JPN J CANCER RES, V77, P682	44
UEDA K, 1987, J BIOL CHEM	UEDA K, 1987, J BIOL CHEM, V262, P505	44
TEWEY KM, 1984, SCIENCE	TEWEY KM, 1984, SCIENCE, V226, P466	44
SAFA AR, 1987, J BIOL CHEM	SAFA AR, 1987, J BIOL CHEM, V262, P7884	44
SEEBER S, 1982, CANCER RES	SEEBER S, 1982, CANCER RES, V42, P4719	44
DOLNICK BJ, 1979, J CELL BIOL	DOLNICK BJ, 1979, J CELL BIOL, V83, P394	43
BECK WT, 1986, CANCER RES	BECK WT, 1986, CANCER RES, V46, P778	43
OZOLS RF, 1987, J CLIN ONCOL	OZOLS RF, 1987, J CLIN ONCOL, V5, P641	42
RAMU A, 1983, CANCER RES	RAMU A, 1983, CANCER RES, V43, P5533	42
WILLINGHAM MC, 1986, CANCER RES	WILLINGHAM MC, 1986, CANCER RES, V46, P5941	42
SKOVSGAARD T, 1978, BIOCHEM PHARMACOL	SKOVSGAARD T, 1978, BIOCHEM PHARMACOL, V27, P1221	42
KAHN CR, 1976, NEW ENGL J MED	KAHN CR, 1976, NEW ENGL J MED, V294, P739	41
TSURUO T, 1983, CANCER TREAT REP	TSURUO T, 1983, CANCER TREAT REP, V67, P889	41
DANKS MK, 1987, CANCER RES	DANKS MK, 1987, CANCER RES, V47, P1297	41
Maniatis T., 1982, MOL CLONING	Maniatis T., 1982, MOL CLONING	41
CURT GA, 1983, NEW ENGL J MED	CURT GA, 1983, NEW ENGL J MED, V308, P199	41
SIROTNAK FM, 1981, CANCER RES	SIROTNAK FM, 1981, CANCER RES, V41, P4447	41
FOJO AT, 1987, J CLIN ONCOL	FOJO AT, 1987, J CLIN ONCOL, V5, P1922	41

Schabel F M Jr, 1978, Antibiot Chemother	Schabel F M Jr, 1978, Antibiot Chemother, V23, P200	41
SALMON SE, 1980, CLONING HUMAN TUMOR	SALMON SE, 1980, CLONING HUMAN TUMOR, P223	40
SCHIMKE RT, 1978, SCIENCE	SCHIMKE RT, 1978, SCIENCE, V202, P1051	40
CHU MY, 1968, BIOCHEM PHARMACOL	CHU MY, 1968, BIOCHEM PHARMACOL, V17, P753	40
DANO K, 1971, CANCER CHEMOTH REP 1	DANO K, 1971, CANCER CHEMOTH REP 1, V55, P133	40
RAMU A, 1984, BRIT J CANCER	RAMU A, 1984, BRIT J CANCER, V50, P501	40
BIEDLER JL, 1975, J NATL CANCER I	BIEDLER JL, 1975, J NATL CANCER I, V55, P671	40
KAYE S, 1985, CANCER CHEMOTH PHARM	KAYE S, 1985, CANCER CHEMOTH PHARM, V14, P96	39
VOLM M, 1979, EUR J CANCER	VOLM M, 1979, EUR J CANCER, V15, P983	39
WAHL GM, 1979, J BIOL CHEM	WAHL GM, 1979, J BIOL CHEM, V254, P8679	39
BIEDLER JL, 1976, SCIENCE	BIEDLER JL, 1976, SCIENCE, V191, P185	39
GREEN JA, 1984, CANCER RES	GREEN JA, 1984, CANCER RES, V44, P5427	39
INABA M, 1979, CANCER LETT	INABA M, 1979, CANCER LETT, V8, P111	38
ZWELLING LA, 1981, CANCER RES	ZWELLING LA, 1981, CANCER RES, V41, P640	38
SELBY P, 1983, NEW ENGL J MED	SELBY P, 1983, NEW ENGL J MED, V308, P129	38
FAIRCHILD CR, 1987, CANCER RES	FAIRCHILD CR, 1987, CANCER RES, V47, P5141	38
BECK WT, 1987, CANCER RES	BECK WT, 1987, CANCER RES, V47, P5455	38
NOWELL PC, 1976, SCIENCE	NOWELL PC, 1976, SCIENCE, V194, P23	38
KAPLAN EL, 1958, J AM STAT ASSOC	KAPLAN EL, 1958, J AM STAT ASSOC, V53, P457	38
BECK WT, 1983, MOL PHARMACOL	BECK WT, 1983, MOL PHARMACOL, V24, P485	37
RICHON VM, 1987, CANCER RES	RICHON VM, 1987, CANCER RES, V47, P2056	37
VONHOFF DD, 1983, CANCER RES	VONHOFF DD, 1983, CANCER RES, V43, P1926	37
BEHRENS BC, 1987, CANCER RES	BEHRENS BC, 1987, CANCER RES, V47, P414	37
RIEHM H, 1972, CANCER RES	RIEHM H, 1972, CANCER RES, V32, P1195	37
MEYERS MB, 1981, BIOCHEM BIOPH RES CO	MEYERS MB, 1981, BIOCHEM BIOPH RES CO, V99, P228	37
BHALLA K, 1985, CANCER RES	BHALLA K, 1985, CANCER RES, V45, P3657	36
GIAVAZZI R, 1983, CANCER RES	GIAVAZZI R, 1983, CANCER RES, V43, P2216	36
ARRICK BA, 1984, CANCER RES	ARRICK BA, 1984, CANCER RES, V44, P4224	35
HABIG WH, 1974, J BIOL CHEM	HABIG WH, 1974, J BIOL CHEM, V249, P7130	35
VANDERBLIEK AM, 1987, EMBO J	VANDERBLIEK AM, 1987, EMBO J, V6, P3325	35
BAKER RM, 1974, CELL	BAKER RM, 1974, CELL, V1, P9	35
NIETHAMMER D, 1975, EUR J CANCER	NIETHAMMER D, 1975, EUR J CANCER, V11, P845	34
DALTON WS, 1986, CANCER RES	DALTON WS, 1986, CANCER RES, V46, P5125	34
HILL BT, 1979, CANCER RES	HILL BT, 1979, CANCER RES, V39, P2440	34
HAKALA MT, 1961, J BIOL CHEM	HAKALA MT, 1961, J BIOL CHEM, V236, P952	34
SKOVSGAARD T, 1980, CANCER RES	SKOVSGAARD T, 1980, CANCER RES, V40, P1077	34
SCHABEL FM, 1983, CANCER TREAT REP	SCHABEL FM, 1983, CANCER TREAT REP, V67, P905	34
SKIPPER H E, 1964, Cancer Chemother Rep	SKIPPER H E, 1964, Cancer Chemother Rep, V35, P1	34
JACKSON RC, 1975, CANCER BIOCHEM BIOPH	JACKSON RC, 1975, CANCER BIOCHEM BIOPH, V1, P151	33
HABER DA, 1981, J BIOL CHEM	HABER DA, 1981, J BIOL CHEM, V256, P9501	33
DURIE BGM, 1975, CANCER	DURIE BGM, 1975, CANCER, V36, P842	33
MIRSKI SEL, 1987, CANCER RES	MIRSKI SEL, 1987, CANCER RES, V47, P2594	33
DEVITA VT, 1983, CANCER	DEVITA VT, 1983, CANCER, V51, P1209	33
WANG AL, 1985, CANCER TREAT REP	WANG AL, 1985, CANCER TREAT REP, V69, P677	33
THOMAS PS, 1980, P NATL ACAD SCI-BIOL	THOMAS PS, 1980, P NATL ACAD SCI-BIOL, V77, P5201	33
SCHIMKE RT, 1984, CANCER RES	SCHIMKE RT, 1984, CANCER RES, V44, P1735	33
HAMADA H, 1988, J BIOL CHEM	HAMADA H, 1988, J BIOL CHEM, V263, P1454	33
FISCHER GA, 1962, BIOCHEM PHARMACOL	FISCHER GA, 1962, BIOCHEM PHARMACOL, V11, P1233	32
MELERA PW, 1980, J BIOL CHEM	MELERA PW, 1980, J BIOL CHEM, V255, P7024	32
FREI E, 1985, P NATL ACAD SCI USA	FREI E, 1985, P NATL ACAD SCI USA, V82, P2158	32

FOJO AT, 1985, P NATL ACAD SCI USA	FOJO AT, 1985, P NATL ACAD SCI USA, V82, P7661	32
MOSMANN T, 1983, J IMMUNOL METHODS	MOSMANN T, 1983, J IMMUNOL METHODS, V65, P55	32
KRAMER RA, 1988, SCIENCE	KRAMER RA, 1988, SCIENCE, V241, P694	32
SIROTNAK FM, 1968, CANCER RES	SIROTNAK FM, 1968, CANCER RES, V28, P75	32
CHEN TR, 1977, EXP CELL RES	CHEN TR, 1977, EXP CELL RES, V104, P255	32
HAMBURGER AW, 1978, CANCER RES	HAMBURGER AW, 1978, CANCER RES, V38, P3438	32
PFAHL M, 1978, MOL CELL ENDOCRINOL	PFAHL M, 1978, MOL CELL ENDOCRINOL, V10, P193	32
OZOLS RF, 1984, SEMIN ONCOL	OZOLS RF, 1984, SEMIN ONCOL, V11, P251	31
THORGEIRSSON SS, 1987, SCIENCE	THORGEIRSSON SS, 1987, SCIENCE, V236, P1120	31
KESSEL D, 1984, BIOCHEM PHARMACOL	KESSEL D, 1984, BIOCHEM PHARMACOL, V33, P1157	31
ALT FW, 1976, J BIOL CHEM	ALT FW, 1976, J BIOL CHEM, V251, P3063	31
FLINTOFF WF, 1980, BIOCHEMISTRY-US	FLINTOFF WF, 1980, BIOCHEMISTRY-US, V19, P4321	31
BURTON K, 1956, BIOCHEM J	BURTON K, 1956, BIOCHEM J, V62, P315	31
RIGBY PWJ, 1977, J MOL BIOL	RIGBY PWJ, 1977, J MOL BIOL, V113, P237	31
GANAPATHI R, 1982, J NATL CANCER I	GANAPATHI R, 1982, J NATL CANCER I, V68, P1027	31
ERICKSON LC, 1980, NATURE	ERICKSON LC, 1980, NATURE, V288, P727	30
SCHIMKE RT, 1984, CELL	SCHIMKE RT, 1984, CELL, V37, P705	30
HAMBURGER A, 1977, J CLIN INVEST	HAMBURGER A, 1977, J CLIN INVEST, V60, P846	30
JACKSON RC, 1977, EUR J CANCER	JACKSON RC, 1977, EUR J CANCER, V13, P567	30
PETERSON RHF, 1978, J SUPRAMOL STR CELL	PETERSON RHF, 1978, J SUPRAMOL STR CELL, V9, P289	30
HORNS RC, 1984, J CLIN ONCOL	HORNS RC, 1984, J CLIN ONCOL, V2, P2	30
WHEELER C, 1982, BIOCHEM PHARMACOL	WHEELER C, 1982, BIOCHEM PHARMACOL, V31, P2691	30

Top 200 cited references during the 1995-2010 period

Short reference (on maps)	Detailed reference	Nr. of citations
COLE SPC, 1992, SCIENCE	COLE SPC, 1992, SCIENCE, V258, P1650	634
GOTTESMAN MM, 1993, ANNU REV BIOCHEM	GOTTESMAN MM, 1993, ANNU REV BIOCHEM, V62, P385	544
Druker BJ, 2001, NEW ENGL J MED	Druker BJ, 2001, NEW ENGL J MED, V344, P1031	523
Gorre ME, 2001, SCIENCE	Gorre ME, 2001, SCIENCE, V293, P876	503
Gottesman MM, 2002, NAT REV CANCER	Gottesman MM, 2002, NAT REV CANCER, V2, P48	475
Lynch TJ, 2004, NEW ENGL J MED	Lynch TJ, 2004, NEW ENGL J MED, V350, P2129	366
MOSMANN T, 1983, J IMMUNOL METHODS	MOSMANN T, 1983, J IMMUNOL METHODS, V65, P55	365
Shah NP, 2002, CANCER CELL	Shah NP, 2002, CANCER CELL, V2, P117	327
Doyle LA, 1998, P NATL ACAD SCI USA	Doyle LA, 1998, P NATL ACAD SCI USA, V95, P15665	326
Therasse P, 2000, J NATL CANCER I	Therasse P, 2000, J NATL CANCER I, V92, P205	323
Paez JG, 2004, SCIENCE	Paez JG, 2004, SCIENCE, V304, P1497	320
KAPLAN EL, 1958, J AM STAT ASSOC	KAPLAN EL, 1958, J AM STAT ASSOC, V53, P457	311
JULIANO RL, 1976, BIOCHIM BIOPHYS ACTA	JULIANO RL, 1976, BIOCHIM BIOPHYS ACTA, V455, P152	285
Pao W, 2005, PLOS MED	Pao W, 2005, PLOS MED, V2, P225	285
ENDICOTT JA, 1989, ANNU REV BIOCHEM	ENDICOTT JA, 1989, ANNU REV BIOCHEM, V58, P137	282
SKEHAN P, 1990, J NATL CANCER I	SKEHAN P, 1990, J NATL CANCER I, V82, P1107	277
BRADFORD MM, 1976, ANAL BIOCHEM	BRADFORD MM, 1976, ANAL BIOCHEM, V72, P248	266
LOWE SW, 1993, CELL	LOWE SW, 1993, CELL, V74, P957	264
Druker BJ, 1996, NAT MED	Druker BJ, 1996, NAT MED, V2, P561	259
TSURUO T, 1981, CANCER RES	TSURUO T, 1981, CANCER RES, V41, P1967	258
CHOMCZYNSKI P, 1987, ANAL BIOCHEM	CHOMCZYNSKI P, 1987, ANAL BIOCHEM, V162, P156	254
Hanahan D, 2000, CELL	Hanahan D, 2000, CELL, V100, P57	250
Shah NP, 2004, SCIENCE	Shah NP, 2004, SCIENCE, V305, P399	244
Slamon DJ, 2001, NEW ENGL J MED	Slamon DJ, 2001, NEW ENGL J MED, V344, P783	228
O'Brien SG, 2003, NEW ENGL J MED	O'Brien SG, 2003, NEW ENGL J MED, V348, P994	223
CHOU TC, 1984, ADV ENZYME REGUL	CHOU TC, 1984, ADV ENZYME REGUL, V22, P27	207
Borst P, 2000, J NATL CANCER I	Borst P, 2000, J NATL CANCER I, V92, P1295	205
GRANT CE, 1994, CANCER RES	GRANT CE, 1994, CANCER RES, V54, P357	205
Kantarjian H, 2002, NEW ENGL J MED	Kantarjian H, 2002, NEW ENGL J MED, V346, P645	205
Kobayashi S, 2005, NEW ENGL J MED	Kobayashi S, 2005, NEW ENGL J MED, V352, P786	204
McGuire WP, 1996, NEW ENGL J MED	McGuire WP, 1996, NEW ENGL J MED, V334, P1	203
Hochhaus A, 2002, LEUKEMIA	Hochhaus A, 2002, LEUKEMIA, V16, P2190	201
SLAMON DJ, 1987, SCIENCE	SLAMON DJ, 1987, SCIENCE, V235, P177	200
COLE SPC, 1994, CANCER RES	COLE SPC, 1994, CANCER RES, V54, P5902	199
GOLDSTEIN LJ, 1989, J NATL CANCER I	GOLDSTEIN LJ, 1989, J NATL CANCER I, V81, P116	199
ZAMAN GJR, 1994, P NATL ACAD SCI USA	ZAMAN GJR, 1994, P NATL ACAD SCI USA, V91, P8822	196
Mahon FX, 2000, BLOOD	Mahon FX, 2000, BLOOD, V96, P1070	193
CHEN CJ, 1986, CELL	CHEN CJ, 1986, CELL, V47, P381	193
Pao W, 2004, P NATL ACAD SCI USA	Pao W, 2004, P NATL ACAD SCI USA, V101, P13306	187
SCHEPER RJ, 1993, CANCER RES	SCHEPER RJ, 1993, CANCER RES, V53, P1475	187
FORD JM, 1990, PHARMACOL REV	FORD JM, 1990, PHARMACOL REV, V42, P155	182
Szakacs G, 2006, NAT REV DRUG DISCOV	Szakacs G, 2006, NAT REV DRUG DISCOV, V5, P219	181
Weisberg E, 2005, CANCER CELL	Weisberg E, 2005, CANCER CELL, V7, P129	181
MILLER AB, 1981, CANCER	MILLER AB, 1981, CANCER, V47, P207	180
Schindler T, 2000, SCIENCE	Schindler T, 2000, SCIENCE, V289, P1938	179

Siddik ZH, 2003, ONCOGENE	Siddik ZH, 2003, ONCOGENE, V22, P7265	178
SCHEFFER GL, 1995, NAT MED	SCHEFFER GL, 1995, NAT MED, V1, P578	178
Druker BJ, 2006, NEW ENGL J MED	Druker BJ, 2006, NEW ENGL J MED, V355, P2408	178
Branford S, 2002, BLOOD	Branford S, 2002, BLOOD, V99, P3472	177
Talpaz M, 2006, NEW ENGL J MED	Talpaz M, 2006, NEW ENGL J MED, V354, P2531	173
Engelman JA, 2007, SCIENCE	Engelman JA, 2007, SCIENCE, V316, P1039	173
Al-Hajj M, 2003, P NATL ACAD SCI USA	Al-Hajj M, 2003, P NATL ACAD SCI USA, V100, P3983	171
Tannock IF, 2004, NEW ENGL J MED	Tannock IF, 2004, NEW ENGL J MED, V351, P1502	170
FOJO AT, 1987, P NATL ACAD SCI USA	FOJO AT, 1987, P NATL ACAD SCI USA, V84, P265	170
Sawyers CL, 2002, BLOOD	Sawyers CL, 2002, BLOOD, V99, P3530	170
LAEMMLI UK, 1970, NATURE	LAEMMLI UK, 1970, NATURE, V227, P680	167
Miyake K, 1999, CANCER RES	Miyake K, 1999, CANCER RES, V59, P8	167
CARMICHAEL J, 1987, CANCER RES	CARMICHAEL J, 1987, CANCER RES, V47, P936	166
Branford S, 2003, BLOOD	Branford S, 2003, BLOOD, V102, P276	163
Ambudkar SV, 1999, ANNU REV PHARMACOL	Ambudkar SV, 1999, ANNU REV PHARMACOL, V39, P361	162
Dean M, 2005, NAT REV CANCER	Dean M, 2005, NAT REV CANCER, V5, P275	161
TEW KD, 1994, CANCER RES	TEW KD, 1994, CANCER RES, V54, P4313	160
Nagata Y, 2004, CANCER CELL	Nagata Y, 2004, CANCER CELL, V6, P117	159
Donato NJ, 2003, BLOOD	Donato NJ, 2003, BLOOD, V101, P690	158
Fukuoka M, 2003, J CLIN ONCOL	Fukuoka M, 2003, J CLIN ONCOL, V21, P2237	157
GODWIN AK, 1992, P NATL ACAD SCI USA	GODWIN AK, 1992, P NATL ACAD SCI USA, V89, P3070	155
Giannakakou P, 1997, J BIOL CHEM	Giannakakou P, 1997, J BIOL CHEM, V272, P17118	154
SLAMON DJ, 1989, SCIENCE	SLAMON DJ, 1989, SCIENCE, V244, P707	153
Gottesman MM, 2002, ANNU REV MED	Gottesman MM, 2002, ANNU REV MED, V53, P615	151
Kool M, 1997, CANCER RES	Kool M, 1997, CANCER RES, V57, P3537	150
LOWE SW, 1994, SCIENCE	LOWE SW, 1994, SCIENCE, V266, P807	149
Johnstone RW, 2002, CELL	Johnstone RW, 2002, CELL, V108, P153	149
von Bubnoff N, 2002, LANCET	von Bubnoff N, 2002, LANCET, V359, P487	148
Kris MG, 2003, JAMA-J AM MED ASSOC	Kris MG, 2003, JAMA-J AM MED ASSOC, V290, P2149	147
Allikmets R, 1998, CANCER RES	Allikmets R, 1998, CANCER RES, V58, P5337	146
Shou J, 2004, J NATL CANCER I	Shou J, 2004, J NATL CANCER I, V96, P926	146
Pan GH, 1997, SCIENCE	Pan GH, 1997, SCIENCE, V277, P815	145
Talpaz M, 2002, BLOOD	Talpaz M, 2002, BLOOD, V99, P1928	144
Petrylak DP, 2004, NEW ENGL J MED	Petrylak DP, 2004, NEW ENGL J MED, V351, P1513	144
O'Hare T, 2005, CANCER RES	O'Hare T, 2005, CANCER RES, V65, P4500	142
Roche-Lestienne C, 2002, BLOOD	Roche-Lestienne C, 2002, BLOOD, V100, P1014	141
THIEBAUT F, 1987, P NATL ACAD SCI USA	THIEBAUT F, 1987, P NATL ACAD SCI USA, V84, P7735	139
Demetri GD, 2002, NEW ENGL J MED	Demetri GD, 2002, NEW ENGL J MED, V347, P472	139
Cunningham D, 2004, NEW ENGL J MED	Cunningham D, 2004, NEW ENGL J MED, V351, P337	137
le Coutre P, 2000, BLOOD	le Coutre P, 2000, BLOOD, V95, P1758	135
Shepherd FA, 2005, NEW ENGL J MED	Shepherd FA, 2005, NEW ENGL J MED, V353, P123	134
Kantarjian H, 2006, NEW ENGL J MED	Kantarjian H, 2006, NEW ENGL J MED, V354, P2542	134
UEDA K, 1987, P NATL ACAD SCI USA	UEDA K, 1987, P NATL ACAD SCI USA, V84, P3004	134
CHAUDHARY PM, 1991, CELL	CHAUDHARY PM, 1991, CELL, V66, P85	133
Jemal A, 2008, CA-CANCER J CLIN	Jemal A, 2008, CA-CANCER J CLIN, V58, P71	132
Ashkenazi A, 1999, J CLIN INVEST	Ashkenazi A, 1999, J CLIN INVEST, V104, P155	132
Bonnet D, 1997, NAT MED	Bonnet D, 1997, NAT MED, V3, P730	130
Hurwitz H, 2004, NEW ENGL J MED	Hurwitz H, 2004, NEW ENGL J MED, V350, P2335	128
Vogel CL, 2002, J CLIN ONCOL	Vogel CL, 2002, J CLIN ONCOL, V20, P719	126
Azam M, 2003, CELL	Azam M, 2003, CELL, V112, P831	126
NICOLETTI I, 1991, J IMMUNOL METHODS	NICOLETTI I, 1991, J IMMUNOL METHODS, V139, P271	126
Zhou S, 2001, NAT MED	Zhou S, 2001, NAT MED, V7, P1028	125
ANDREWS PA, 1990, CANCER CELL-	ANDREWS PA, 1990, CANCER CELL-MON REV, V2,	124

MON REV	P35	
DALEY GQ, 1990, SCIENCE	DALEY GQ, 1990, SCIENCE, V247, P824	123
Sordella R, 2004, SCIENCE	Sordella R, 2004, SCIENCE, V305, P1163	123
Singh SK, 2004, NATURE	Singh SK, 2004, NATURE, V432, P396	123
Walczak H, 1999, NAT MED	Walczak H, 1999, NAT MED, V5, P157	123
Reya T, 2001, NATURE	Reya T, 2001, NATURE, V414, P105	123
Tannock IF, 1996, J CLIN ONCOL	Tannock IF, 1996, J CLIN ONCOL, V14, P1756	122
MULLER M, 1994, P NATL ACAD SCI USA	MULLER M, 1994, P NATL ACAD SCI USA, V91, P13033	121
Maliepaard M, 1999, CANCER RES	Maliepaard M, 1999, CANCER RES, V59, P4559	120
NOONAN KE, 1990, P NATL ACAD SCI USA	NOONAN KE, 1990, P NATL ACAD SCI USA, V87, P7160	119
Lu YH, 2001, J NATL CANCER I	Lu YH, 2001, J NATL CANCER I, V93, P1852	118
CAMPOS L, 1992, BLOOD	CAMPOS L, 1992, BLOOD, V79, P473	117
GATELY DP, 1993, BRIT J CANCER	GATELY DP, 1993, BRIT J CANCER, V67, P1171	116
Doyle LA, 2003, ONCOGENE	Doyle LA, 2003, ONCOGENE, V22, P7340	116
KELLEY SL, 1988, SCIENCE	KELLEY SL, 1988, SCIENCE, V241, P1813	116
Maliepaard M, 2001, CANCER RES	Maliepaard M, 2001, CANCER RES, V61, P3458	116
Perou CM, 2000, NATURE	Perou CM, 2000, NATURE, V406, P747	116
Deininger M, 2005, BLOOD	Deininger M, 2005, BLOOD, V105, P2640	115
MARKMAN M, 1991, J CLIN ONCOL	MARKMAN M, 1991, J CLIN ONCOL, V9, P389	114
Nagar B, 2002, CANCER RES	Nagar B, 2002, CANCER RES, V62, P4236	114
Kavallaris M, 1997, J CLIN INVEST	Kavallaris M, 1997, J CLIN INVEST, V100, P1282	114
Kartalou M, 2001, MUTAT RES-FUND MOL M	Kartalou M, 2001, MUTAT RES-FUND MOL M, V478, P23	113
KARTNER N, 1983, SCIENCE	KARTNER N, 1983, SCIENCE, V221, P1285	112
Krishna R, 2000, EUR J PHARM SCI	Krishna R, 2000, EUR J PHARM SCI, V11, P265	112
Hughes TP, 2003, NEW ENGL J MED	Hughes TP, 2003, NEW ENGL J MED, V349, P1423	112
MIYASHITA T, 1995, CELL	MIYASHITA T, 1995, CELL, V80, P293	111
Weisberg E, 2000, BLOOD	Weisberg E, 2000, BLOOD, V95, P3498	111
FISHER DE, 1994, CELL	FISHER DE, 1994, CELL, V78, P539	111
Ambudkar SV, 2003, ONCOGENE	Ambudkar SV, 2003, ONCOGENE, V22, P7468	110
Heinrich MC, 2003, J CLIN ONCOL	Heinrich MC, 2003, J CLIN ONCOL, V21, P4342	110
ZAMAN GJR, 1995, P NATL ACAD SCI USA	ZAMAN GJR, 1995, P NATL ACAD SCI USA, V92, P7690	110
Borst P, 2002, ANNU REV BIOCHEM	Borst P, 2002, ANNU REV BIOCHEM, V71, P537	110
Loe DW, 1996, J BIOL CHEM	Loe DW, 1996, J BIOL CHEM, V271, P9675	110
Fink D, 1996, CANCER RES	Fink D, 1996, CANCER RES, V56, P4881	109
Scherf U, 2000, NAT GENET	Scherf U, 2000, NAT GENET, V24, P236	109
ALLEY MC, 1988, CANCER RES	ALLEY MC, 1988, CANCER RES, V48, P589	109
LIU LF, 1989, ANNU REV BIOCHEM	LIU LF, 1989, ANNU REV BIOCHEM, V58, P351	109
LOWRY OH, 1951, J BIOL CHEM	LOWRY OH, 1951, J BIOL CHEM, V193, P265	109
Richardson PG, 2003, NEW ENGL J MED	Richardson PG, 2003, NEW ENGL J MED, V348, P2609	108
LEIER I, 1994, J BIOL CHEM	LEIER I, 1994, J BIOL CHEM, V269, P27807	108
Leith CP, 1999, BLOOD	Leith CP, 1999, BLOOD, V94, P1086	108
Ross DD, 1999, J NATL CANCER I	Ross DD, 1999, J NATL CANCER I, V91, P429	108
Loe D. W., 1996, European Journal of Cancer	Loe D. W., 1996, European Journal of Cancer, V32A, P945	108
Buchdunger E, 1996, CANCER RES	Buchdunger E, 1996, CANCER RES, V56, P100	108
ISHIKAWA T, 1993, J BIOL CHEM	ISHIKAWA T, 1993, J BIOL CHEM, V268, P20116	108
Knowlden JM, 2003, ENDOCRINOLOGY	Knowlden JM, 2003, ENDOCRINOLOGY, V144, P1032	108
Taniguchi K, 1996, CANCER RES	Taniguchi K, 1996, CANCER RES, V56, P4124	107
Beck WT, 1996, CANCER RES	Beck WT, 1996, CANCER RES, V56, P3010	107
Litman T, 2001, CELL MOL LIFE SCI	Litman T, 2001, CELL MOL LIFE SCI, V58, P931	107
Clarke M, 1998, LANCET	Clarke M, 1998, LANCET, V351, P1451	106
Sorlie T, 2001, P NATL ACAD SCI USA	Sorlie T, 2001, P NATL ACAD SCI USA, V98, P10869	106
Mahon FX, 2003, BLOOD	Mahon FX, 2003, BLOOD, V101, P2368	105
Yarden Y, 2001, NAT REV MOL CELL BIO	Yarden Y, 2001, NAT REV MOL CELL BIO, V2, P127	105
Cobleigh MA, 1999, J CLIN ONCOL	Cobleigh MA, 1999, J CLIN ONCOL, V17, P2639	105
Campbell RA, 2001, J BIOL CHEM	Campbell RA, 2001, J BIOL CHEM, V276, P9817	105

LIST AF, 1993, J CLIN ONCOL	LIST AF, 1993, J CLIN ONCOL, V11, P1652	104
BATIST G, 1986, J BIOL CHEM	BATIST G, 1986, J BIOL CHEM, V261, P5544	104
Graham SM, 2002, BLOOD	Graham SM, 2002, BLOOD, V99, P319	104
Friesen C, 1996, NAT MED	Friesen C, 1996, NAT MED, V2, P574	103
Kwak EL, 2005, P NATL ACAD SCI USA	Kwak EL, 2005, P NATL ACAD SCI USA, V102, P7665	103
MIYASHITA T, 1993, BLOOD	MIYASHITA T, 1993, BLOOD, V81, P151	103
OLTVAI ZN, 1993, CELL	OLTVAI ZN, 1993, CELL, V74, P609	103
PASTAN I, 1987, NEW ENGL J MED	PASTAN I, 1987, NEW ENGL J MED, V316, P1388	103
Sambrook J, 1989, MOL CLONING LAB MANU	Sambrook J, 1989, MOL CLONING LAB MANU	103
Li P, 1997, CELL	Li P, 1997, CELL, V91, P479	102
SCHNEIDER E, 1994, CANCER RES	SCHNEIDER E, 1994, CANCER RES, V54, P152	101
HYAFIL F, 1993, CANCER RES	HYAFIL F, 1993, CANCER RES, V53, P4595	101
Baccarani M, 2006, BLOOD	Baccarani M, 2006, BLOOD, V108, P1809	101
SCHINKEL AH, 1994, CELL	SCHINKEL AH, 1994, CELL, V77, P491	101
Levine AJ, 1997, CELL	Levine AJ, 1997, CELL, V88, P323	100
Wiley SR, 1995, IMMUNITY	Wiley SR, 1995, IMMUNITY, V3, P673	99
Damiano JS, 1999, BLOOD	Damiano JS, 1999, BLOOD, V93, P1658	99
Wang CY, 1996, SCIENCE	Wang CY, 1996, SCIENCE, V274, P784	98
HIGGINS CF, 1992, ANNU REV CELL BIOL	HIGGINS CF, 1992, ANNU REV CELL BIOL, V8, P67	98
Jordan MA, 2004, NAT REV CANCER	Jordan MA, 2004, NAT REV CANCER, V4, P253	98
Thomas Hilary, 2003, Cancer Control	Thomas Hilary, 2003, Cancer Control, V10, P159	98
GROS P, 1986, NATURE	GROS P, 1986, NATURE, V323, P728	98
Irmler M, 1997, NATURE	Irmler M, 1997, NATURE, V388, P190	98
Ashkenazi A, 1998, SCIENCE	Ashkenazi A, 1998, SCIENCE, V281, P1305	98
Bubley GJ, 1999, J CLIN ONCOL	Bubley GJ, 1999, J CLIN ONCOL, V17, P3461	98
BRADLEY G, 1988, BIOCHIM BIOPHYS ACTA	BRADLEY G, 1988, BIOCHIM BIOPHYS ACTA, V948, P87	97
Jedlitschky G, 1996, CANCER RES	Jedlitschky G, 1996, CANCER RES, V56, P988	97
CHU G, 1994, J BIOL CHEM	CHU G, 1994, J BIOL CHEM, V269, P787	97
Schiller JH, 2002, NEW ENGL J MED	Schiller JH, 2002, NEW ENGL J MED, V346, P92	97
Gordon AN, 2001, J CLIN ONCOL	Gordon AN, 2001, J CLIN ONCOL, V19, P3312	97
SONNEVELD P, 1992, LANCET	SONNEVELD P, 1992, LANCET, V340, P255	97
SIMON SM, 1994, P NATL ACAD SCI USA	SIMON SM, 1994, P NATL ACAD SCI USA, V91, P3497	97
Vivanco I, 2002, NAT REV CANCER	Vivanco I, 2002, NAT REV CANCER, V2, P489	96
Wang CY, 1999, NAT MED	Wang CY, 1999, NAT MED, V5, P412	96
Sawyers CL, 1999, NEW ENGL J MED	Sawyers CL, 1999, NEW ENGL J MED, V340, P1330	96
Scaffidi C, 1998, EMBO J	Scaffidi C, 1998, EMBO J, V17, P1675	96
Aebi S, 1996, CANCER RES	Aebi S, 1996, CANCER RES, V56, P3087	96
Fink D, 1998, CLIN CANCER RES	Fink D, 1998, CLIN CANCER RES, V4, P1	96
Ciardiello F, 2000, CLIN CANCER RES	Ciardiello F, 2000, CLIN CANCER RES, V6, P2053	95
Osborne CK, 2003, J NATL CANCER I	Osborne CK, 2003, J NATL CANCER I, V95, P353	95
KATO S, 1995, SCIENCE	KATO S, 1995, SCIENCE, V270, P1491	95
Stupp R, 2005, NEW ENGL J MED	Stupp R, 2005, NEW ENGL J MED, V352, P987	95
Romond EH, 2005, NEW ENGL J MED	Romond EH, 2005, NEW ENGL J MED, V353, P1673	95
KASTAN MB, 1991, CANCER RES	KASTAN MB, 1991, CANCER RES, V51, P6304	95
Burris HA, 1997, J CLIN ONCOL	Burris HA, 1997, J CLIN ONCOL, V15, P2403	95
KRISHNAMACHARY N, 1993, CANCER RES	KRISHNAMACHARY N, 1993, CANCER RES, V53, P3658	95
CHIN KV, 1992, SCIENCE	CHIN KV, 1992, SCIENCE, V255, P459	95
Carter TA, 2005, P NATL ACAD SCI USA	Carter TA, 2005, P NATL ACAD SCI USA, V102, P11011	94

Top 200 cited references during the 2010-2012 period

Short reference (on maps)	Detailed reference	Nr. of citations
Therasse P, 2000, J NATL CANCER I	Therasse P, 2000, J NATL CANCER I, V92, P205	196
Gottesman MM, 2002, NAT REV CANCER	Gottesman MM, 2002, NAT REV CANCER, V2, P48	196
Pao W, 2005, PLOS MED	Pao W, 2005, PLOS MED, V2, pe73	186
Engelman JA, 2007, SCIENCE	Engelman JA, 2007, SCIENCE, V316, P1039	175
Szakacs G, 2006, NAT REV DRUG DISCOV	Szakacs G, 2006, NAT REV DRUG DISCOV, V5, P219	152
Gorre ME, 2001, SCIENCE	Gorre ME, 2001, SCIENCE, V293, P876	148
Lynch TJ, 2004, NEW ENGL J MED	Lynch TJ, 2004, NEW ENGL J MED, V350, P2129	146
Paez JG, 2004, SCIENCE	Paez JG, 2004, SCIENCE, V304, P1497	134
Jemal A, 2009, CA-CANCER J CLIN	Jemal A, 2009, CA-CANCER J CLIN, V59, P225	133
Al-Hajj M, 2003, P NATL ACAD SCI USA	Al-Hajj M, 2003, P NATL ACAD SCI USA, V100, P3983	125
Kobayashi S, 2005, NEW ENGL J MED	Kobayashi S, 2005, NEW ENGL J MED, V352, P786	122
Dean M, 2005, NAT REV CANCER	Dean M, 2005, NAT REV CANCER, V5, P275	119
Hanahan D, 2000, CELL	Hanahan D, 2000, CELL, V100, P57	119
Druker BJ, 2006, NEW ENGL J MED	Druker BJ, 2006, NEW ENGL J MED, V355, P2408	113
Slamon DJ, 2001, NEW ENGL J MED	Slamon DJ, 2001, NEW ENGL J MED, V344, P783	109
CHOU TC, 1984, ADV ENZYME REGUL	CHOU TC, 1984, ADV ENZYME REGUL, V22, P27	103
Mok TS, 2009, NEW ENGL J MED	Mok TS, 2009, NEW ENGL J MED, V361, P947	101
Jemal A, 2008, CA-CANCER J CLIN	Jemal A, 2008, CA-CANCER J CLIN, V58, P71	98
Jemal A, 2010, CA-CANCER J CLIN	Jemal A, 2010, CA-CANCER J CLIN, V60, P277	97
Nagata Y, 2004, CANCER CELL	Nagata Y, 2004, CANCER CELL, V6, P117	96
Reya T, 2001, NATURE	Reya T, 2001, NATURE, V414, P105	94
Tannock IF, 2004, NEW ENGL J MED	Tannock IF, 2004, NEW ENGL J MED, V351, P1502	93
SLAMON DJ, 1987, SCIENCE	SLAMON DJ, 1987, SCIENCE, V235, P177	89
Weisberg E, 2005, CANCER CELL	Weisberg E, 2005, CANCER CELL, V7, P129	88
Siddik ZH, 2003, ONCOGENE	Siddik ZH, 2003, ONCOGENE, V22, P7265	88
Shah NP, 2002, CANCER CELL	Shah NP, 2002, CANCER CELL, V2, P117	88
Druker BJ, 2001, NEW ENGL J MED	Druker BJ, 2001, NEW ENGL J MED, V344, P1031	87
Karapetis CS, 2008, NEW ENGL J MED	Karapetis CS, 2008, NEW ENGL J MED, V359, P1757	86
Singh SK, 2004, NATURE	Singh SK, 2004, NATURE, V432, P396	86
Gottesman MM, 2002, ANNU REV MED	Gottesman MM, 2002, ANNU REV MED, V53, P615	82
Pao W, 2004, P NATL ACAD SCI USA	Pao W, 2004, P NATL ACAD SCI USA, V101, P13306	82
Shepherd FA, 2005, NEW ENGL J MED	Shepherd FA, 2005, NEW ENGL J MED, V353, P123	80
Bean J, 2007, P NATL ACAD SCI USA	Bean J, 2007, P NATL ACAD SCI USA, V104, P20932	80
Livak KJ, 2001, METHODS	Livak KJ, 2001, METHODS, V25, P402	80
Kelland L, 2007, NAT REV CANCER	Kelland L, 2007, NAT REV CANCER, V7, P573	78
Bao SD, 2006, NATURE	Bao SD, 2006, NATURE, V444, P756	78
Ricci-Vitiani L, 2007, NATURE	Ricci-Vitiani L, 2007, NATURE, V445, P111	77
Bonnet D, 1997, NAT MED	Bonnet D, 1997, NAT MED, V3, P730	77
Berns K, 2007, CANCER CELL	Berns K, 2007, CANCER CELL, V12, P395	75
Perou CM, 2000, NATURE	Perou CM, 2000, NATURE, V406, P747	74
Shah NP, 2004, SCIENCE	Shah NP, 2004, SCIENCE, V305, P399	73
Parkin DM, 2005, CA-CANCER J CLIN	Parkin DM, 2005, CA-CANCER J CLIN, V55, P74	71
Petrylak DP, 2004, NEW ENGL J MED	Petrylak DP, 2004, NEW ENGL J MED, V351, P1513	71
MOSMANN T, 1983, J IMMUNOL METHODS	MOSMANN T, 1983, J IMMUNOL METHODS, V65, P55	70
Kantarjian H, 2006, NEW ENGL J MED	Kantarjian H, 2006, NEW ENGL J MED, V354, P2542	69
Flaherty KT, 2010, NEW ENGL J MED	Flaherty KT, 2010, NEW ENGL J MED, V363, P809	69
Cunningham D, 2004, NEW ENGL J MED	Cunningham D, 2004, NEW ENGL J MED, V351, P337	69
Doyle LA, 1998, P NATL ACAD SCI USA	Doyle LA, 1998, P NATL ACAD SCI USA, V95, P15665	68

O'Hare T, 2005, CANCER RES	O'Hare T, 2005, CANCER RES, V65, P4500	67
O'Brien SG, 2003, NEW ENGL J MED	O'Brien SG, 2003, NEW ENGL J MED, V348, P994	66
O'Brien CA, 2007, NATURE	O'Brien CA, 2007, NATURE, V445, P106	66
Davies H, 2002, NATURE	Davies H, 2002, NATURE, V417, P949	66
Bartel DP, 2004, CELL	Bartel DP, 2004, CELL, V116, P281	65
Di Nicolantonio F, 2008, J CLIN ONCOL	Di Nicolantonio F, 2008, J CLIN ONCOL, V26, P5705	65
Geyer CE, 2006, NEW ENGL J MED	Geyer CE, 2006, NEW ENGL J MED, V355, P2733	65
Yun CH, 2008, P NATL ACAD SCI USA	Yun CH, 2008, P NATL ACAD SCI USA, V105, P2070	64
Mani SA, 2008, CELL	Mani SA, 2008, CELL, V133, P704	64
Abe O, 2005, LANCET	Abe O, 2005, LANCET, V365, P1687	63
Amado RG, 2008, J CLIN ONCOL	Amado RG, 2008, J CLIN ONCOL, V26, P1626	62
Vogel CL, 2002, J CLIN ONCOL	Vogel CL, 2002, J CLIN ONCOL, V20, P719	61
Sorlie T, 2001, P NATL ACAD SCI USA	Sorlie T, 2001, P NATL ACAD SCI USA, V98, P10869	61
Kantarjian HM, 2007, BLOOD	Kantarjian HM, 2007, BLOOD, V110, P3540	61
Hirschmann-Jax C, 2004, P NATL ACAD SCI USA	Hirschmann-Jax C, 2004, P NATL ACAD SCI USA, V101, P14228	60
Baccarani M, 2009, J CLIN ONCOL	Baccarani M, 2009, J CLIN ONCOL, V27, P6041	60
LAPIDOT T, 1994, NATURE	LAPIDOT T, 1994, NATURE, V367, P645	60
Druker BJ, 1996, NAT MED	Druker BJ, 1996, NAT MED, V2, P561	60
Romond EH, 2005, NEW ENGL J MED	Romond EH, 2005, NEW ENGL J MED, V353, P1673	60
Singh SK, 2003, CANCER RES	Singh SK, 2003, CANCER RES, V63, P5821	60
Shou J, 2004, J NATL CANCER I	Shou J, 2004, J NATL CANCER I, V96, P926	60
Talpaz M, 2006, NEW ENGL J MED	Talpaz M, 2006, NEW ENGL J MED, V354, P2531	59
Stupp R, 2005, NEW ENGL J MED	Stupp R, 2005, NEW ENGL J MED, V352, P987	59
Nazarian R, 2010, NATURE	Nazarian R, 2010, NATURE, V468, P973	58
Farmer H, 2005, NATURE	Farmer H, 2005, NATURE, V434, P917	57
KAPLAN EL, 1958, J AM STAT ASSOC	KAPLAN EL, 1958, J AM STAT ASSOC, V53, P457	55
Oltersdorf T, 2005, NATURE	Oltersdorf T, 2005, NATURE, V435, P677	54
Yarden Y, 2001, NAT REV MOL CELL BIO	Yarden Y, 2001, NAT REV MOL CELL BIO, V2, P127	54
Van Cutsem E, 2009, NEW ENGL J MED	Van Cutsem E, 2009, NEW ENGL J MED, V360, P1408	54
Hochhaus A, 2002, LEUKEMIA	Hochhaus A, 2002, LEUKEMIA, V16, P2190	54
O'Hare T, 2007, BLOOD	O'Hare T, 2007, BLOOD, V110, P2242	53
Branford S, 2003, BLOOD	Branford S, 2003, BLOOD, V102, P276	53
Apperley JF, 2007, LANCET ONCOL	Apperley JF, 2007, LANCET ONCOL, V8, P1018	53
Saglio G, 2010, NEW ENGL J MED	Saglio G, 2010, NEW ENGL J MED, V362, P2251	53
JULIANO RL, 1976, BIOCHIM BIOPHYS ACTA	JULIANO RL, 1976, BIOCHIM BIOPHYS ACTA, V455, P152	53
Sharma SV, 2007, NAT REV CANCER	Sharma SV, 2007, NAT REV CANCER, V7, P169	53
Engelman JA, 2008, CLIN CANCER RES	Engelman JA, 2008, CLIN CANCER RES, V14, P2895	53
Li CW, 2007, CANCER RES	Li CW, 2007, CANCER RES, V67, P1030	52
Gupta PB, 2009, CELL	Gupta PB, 2009, CELL, V138, P645	52
Fukuoka M, 2003, J CLIN ONCOL	Fukuoka M, 2003, J CLIN ONCOL, V21, P2237	52
Fong PC, 2009, NEW ENGL J MED	Fong PC, 2009, NEW ENGL J MED, V361, P123	52
Cortes JE, 2010, J CLIN ONCOL	Cortes JE, 2010, J CLIN ONCOL, V28, P392	52
Piccart-Gebhart MJ, 2005, NEW ENGL J MED	Piccart-Gebhart MJ, 2005, NEW ENGL J MED, V353, P1659	52
Hynes NE, 2005, NAT REV CANCER	Hynes NE, 2005, NAT REV CANCER, V5, P341	52
Zhou S, 2001, NAT MED	Zhou S, 2001, NAT MED, V7, P1028	51
Maemondo M, 2010, NEW ENGL J MED	Maemondo M, 2010, NEW ENGL J MED, V362, P2380	51
Wang D, 2005, NAT REV DRUG DISCOV	Wang D, 2005, NAT REV DRUG DISCOV, V4, P307	51
Kwak EL, 2005, P NATL ACAD SCI USA	Kwak EL, 2005, P NATL ACAD SCI USA, V102, P7665	51
Nahta R, 2005, CANCER RES	Nahta R, 2005, CANCER RES, V65, P11118	51
Yang H, 2008, CANCER RES	Yang H, 2008, CANCER RES, V68, P425	50
Johannessen CM, 2010, NATURE	Johannessen CM, 2010, NATURE, V468, P968	50

Burris HA, 1997, J CLIN ONCOL	Burris HA, 1997, J CLIN ONCOL, V15, P2403	50
Soverini S, 2006, CLIN CANCER RES	Soverini S, 2006, CLIN CANCER RES, V12, P7374	50
Hermann PC, 2007, CELL STEM CELL	Hermann PC, 2007, CELL STEM CELL, V1, P313	50
COLE SPC, 1992, SCIENCE	COLE SPC, 1992, SCIENCE, V258, P1650	50
O'Reilly KE, 2006, CANCER RES	O'Reilly KE, 2006, CANCER RES, V66, P1500	50
Hurwitz H, 2004, NEW ENGL J MED	Hurwitz H, 2004, NEW ENGL J MED, V350, P2335	49
Borst P, 2000, J NATL CANCER I	Borst P, 2000, J NATL CANCER I, V92, P1295	49
Ambudkar SV, 1999, ANNU REV PHARMACOL	Ambudkar SV, 1999, ANNU REV PHARMACOL, V39, P361	49
Cortes J, 2007, BLOOD	Cortes J, 2007, BLOOD, V109, P3207	49
Sharma SV, 2010, CELL	Sharma SV, 2010, CELL, V141, P69	48
Li XX, 2008, J NATL CANCER I	Li XX, 2008, J NATL CANCER I, V100, P672	48
Jemal A, 2007, CA-CANCER J CLIN	Jemal A, 2007, CA-CANCER J CLIN, V57, P43	48
Yano S, 2008, CANCER RES	Yano S, 2008, CANCER RES, V68, P9479	48
Lu YH, 2001, J NATL CANCER I	Lu YH, 2001, J NATL CANCER I, V93, P1852	48
Krishna R, 2000, EUR J PHARM SCI	Krishna R, 2000, EUR J PHARM SCI, V11, P265	48
Hochhaus A, 2009, LEUKEMIA	Hochhaus A, 2009, LEUKEMIA, V23, P1054	48
GOTTESMAN MM, 1993, ANNU REV BIOCHEM	GOTTESMAN MM, 1993, ANNU REV BIOCHEM, V62, P385	47
Mahon FX, 2000, BLOOD	Mahon FX, 2000, BLOOD, V96, P1070	47
Cobleigh MA, 1999, J CLIN ONCOL	Cobleigh MA, 1999, J CLIN ONCOL, V17, P2639	47
Bergers G, 2008, NAT REV CANCER	Bergers G, 2008, NAT REV CANCER, V8, P592	47
Sartore-Bianchi A, 2009, CANCER RES	Sartore-Bianchi A, 2009, CANCER RES, V69, P1851	47
Kris MG, 2003, JAMA-J AM MED ASSOC	Kris MG, 2003, JAMA-J AM MED ASSOC, V290, P2149	46
Demetri GD, 2002, NEW ENGL J MED	Demetri GD, 2002, NEW ENGL J MED, V347, P472	46
Maheswaran S, 2008, NEW ENGL J MED	Maheswaran S, 2008, NEW ENGL J MED, V359, P366	46
Kantarjian H, 2010, NEW ENGL J MED	Kantarjian H, 2010, NEW ENGL J MED, V362, P2260	46
Rabik CA, 2007, CANCER TREAT REV	Rabik CA, 2007, CANCER TREAT REV, V33, P9	46
Guix M, 2008, J CLIN INVEST	Guix M, 2008, J CLIN INVEST, V118, P2609	46
Aller SG, 2009, SCIENCE	Aller SG, 2009, SCIENCE, V323, P1718	46
Schiller JH, 2002, NEW ENGL J MED	Schiller JH, 2002, NEW ENGL J MED, V346, P92	45
Mitsudomi T, 2010, LANCET ONCOL	Mitsudomi T, 2010, LANCET ONCOL, V11, P121	45
Hughes T, 2009, J CLIN ONCOL	Hughes T, 2009, J CLIN ONCOL, V27, P4204	45
Ginestier C, 2007, CELL STEM CELL	Ginestier C, 2007, CELL STEM CELL, V1, P555	45
Thomas J, 2004, BLOOD	Thomas J, 2004, BLOOD, V104, P3739	45
Jabbour E, 2006, LEUKEMIA	Jabbour E, 2006, LEUKEMIA, V20, P1767	45
Tol J, 2009, NEW ENGL J MED	Tol J, 2009, NEW ENGL J MED, V360, P563	45
Lievre A, 2006, CANCER RES	Lievre A, 2006, CANCER RES, V66, P3992	45
Knowlden JM, 2003, ENDOCRINOLOGY	Knowlden JM, 2003, ENDOCRINOLOGY, V144, P1032	45
Baccarani M, 2006, BLOOD	Baccarani M, 2006, BLOOD, V108, P1809	45
Heinrich MC, 2008, J CLIN ONCOL	Heinrich MC, 2008, J CLIN ONCOL, V26, P5352	45
Olaussen KA, 2006, NEW ENGL J MED	Olaussen KA, 2006, NEW ENGL J MED, V355, P983	45
Kovalchuk O, 2008, MOL CANCER THER	Kovalchuk O, 2008, MOL CANCER THER, V7, P2152	44
Bryant HE, 2005, NATURE	Bryant HE, 2005, NATURE, V434, P913	44
Turke AB, 2010, CANCER CELL	Turke AB, 2010, CANCER CELL, V17, P77	44
Demetri GD, 2006, LANCET	Demetri GD, 2006, LANCET, V368, P1329	43
Clarke Michael F, 2006, Cancer Res	Clarke Michael F, 2006, Cancer Res, V66, P9339	43
Khambata-Ford S, 2007, J CLIN ONCOL	Khambata-Ford S, 2007, J CLIN ONCOL, V25, P3230	43
Blanke CD, 2008, J CLIN ONCOL	Blanke CD, 2008, J CLIN ONCOL, V26, P626	42
Xia L, 2008, INT J CANCER	Xia L, 2008, INT J CANCER, V123, P372	42
Calin GA, 2006, NAT REV CANCER	Calin GA, 2006, NAT REV CANCER, V6, P857	42
Sharom FJ, 2008, PHARMACOGENOMICS	Sharom FJ, 2008, PHARMACOGENOMICS, V9, P105, 9.1.105	42
Lievre A, 2008, J CLIN ONCOL	Lievre A, 2008, J CLIN ONCOL, V26, P374	42
Donato NJ, 2003, BLOOD	Donato NJ, 2003, BLOOD, V101, P690	41

Hochhaus A, 2007, BLOOD	Hochhaus A, 2007, BLOOD, V109, P2303	41
Musgrove EA, 2009, NAT REV CANCER	Musgrove EA, 2009, NAT REV CANCER, V9, P631	41
Ritter CA, 2007, CLIN CANCER RES	Ritter CA, 2007, CLIN CANCER RES, V13, P4909	41
SLAMON DJ, 1989, SCIENCE	SLAMON DJ, 1989, SCIENCE, V244, P707	41
Villanueva J, 2010, CANCER CELL	Villanueva J, 2010, CANCER CELL, V18, P683	41
Dalerba P, 2007, P NATL ACAD SCI USA	Dalerba P, 2007, P NATL ACAD SCI USA, V104, P10158	41
Paez-Ribes M, 2009, CANCER CELL	Paez-Ribes M, 2009, CANCER CELL, V15, P220	41
Collins AT, 2005, CANCER RES	Collins AT, 2005, CANCER RES, V65, P10946	40
Moore MJ, 2007, J CLIN ONCOL	Moore MJ, 2007, J CLIN ONCOL, V25, P1960	40
Jonker DJ, 2007, NEW ENGL J MED	Jonker DJ, 2007, NEW ENGL J MED, V357, P2040	40
Bollag G, 2010, NATURE	Bollag G, 2010, NATURE, V467, P596	40
Jabbour E, 2008, BLOOD	Jabbour E, 2008, BLOOD, V112, P53	40
Scaltriti M, 2007, J NATL CANCER I	Scaltriti M, 2007, J NATL CANCER I, V99, P628	40
Poulikakos PI, 2010, NATURE	Poulikakos PI, 2010, NATURE, V464, P427	40
Doyle LA, 2003, ONCOGENE	Doyle LA, 2003, ONCOGENE, V22, P7340	39
Hegi ME, 2005, NEW ENGL J MED	Hegi ME, 2005, NEW ENGL J MED, V352, P997	39
Sorlie T, 2003, P NATL ACAD SCI USA	Sorlie T, 2003, P NATL ACAD SCI USA, V100, P8418	39
Chapman PB, 2011, NEW ENGL J MED	Chapman PB, 2011, NEW ENGL J MED, V364, P2507	39
O'Hare T, 2009, CANCER CELL	O'Hare T, 2009, CANCER CELL, V16, P401	39
Longley DB, 2005, J PATHOL	Longley DB, 2005, J PATHOL, V205, P275	39
Motzer RJ, 2007, NEW ENGL J MED	Motzer RJ, 2007, NEW ENGL J MED, V356, P115	39
Soda M, 2007, NATURE	Soda M, 2007, NATURE, V448, P561	39
Stewart DJ, 2007, CRIT REV ONCOL HEMAT	Stewart DJ, 2007, CRIT REV ONCOL HEMAT, V63, P12	39
Azam M, 2003, CELL	Azam M, 2003, CELL, V112, P831	39
Liu GT, 2006, MOL CANCER	Liu GT, 2006, MOL CANCER, V5	39
Wilhelm SM, 2004, CANCER RES	Wilhelm SM, 2004, CANCER RES, V64, P7099	39
Solit DB, 2006, NATURE	Solit DB, 2006, NATURE, V439, P358	38
Jordan MA, 2004, NAT REV CANCER	Jordan MA, 2004, NAT REV CANCER, V4, P253	38
Bradeen HA, 2006, BLOOD	Bradeen HA, 2006, BLOOD, V108, P2332	38
Ho MM, 2007, CANCER RES	Ho MM, 2007, CANCER RES, V67, P4827	38
BRADFORD MM, 1976, ANAL BIOCHEM	BRADFORD MM, 1976, ANAL BIOCHEM, V72, P248	38
Jabbour E, 2009, BLOOD	Jabbour E, 2009, BLOOD, V113, P2154	38
Kantarjian H, 2002, NEW ENGL J MED	Kantarjian H, 2002, NEW ENGL J MED, V346, P645	38
Frattini M, 2007, BRIT J CANCER	Frattini M, 2007, BRIT J CANCER, V97, P1139	38
Muller MC, 2009, BLOOD	Muller MC, 2009, BLOOD, V114, P4944	38
Ambudkar SV, 2003, ONCOGENE	Ambudkar SV, 2003, ONCOGENE, V22, P7468	38
Hanahan D, 2011, CELL	Hanahan D, 2011, CELL, V144, P646	38
Hughes T, 2006, BLOOD	Hughes T, 2006, BLOOD, V108, P28	38
Herbst RS, 2005, J CLIN ONCOL	Herbst RS, 2005, J CLIN ONCOL, V23, P5892	38
Allegra CJ, 2009, J CLIN ONCOL	Allegra CJ, 2009, J CLIN ONCOL, V27, P2091	38
van de Vijver MJ, 2002, NEW ENGL J MED	van de Vijver MJ, 2002, NEW ENGL J MED, V347, P1999	37
Engelman JA, 2006, J CLIN INVEST	Engelman JA, 2006, J CLIN INVEST, V116, P2695	37
Zhu H, 2008, BIOCHEM PHARMACOL	Zhu H, 2008, BIOCHEM PHARMACOL, V76, P582	37
Balak MN, 2006, CLIN CANCER RES	Balak MN, 2006, CLIN CANCER RES, V12, P6494	37
SIMON R, 1989, CONTROL CLIN TRIALS	SIMON R, 1989, CONTROL CLIN TRIALS, V10, P1	37
Konecny GE, 2006, CANCER RES	Konecny GE, 2006, CANCER RES, V66, P1630	37
Agarwal R, 2003, NAT REV CANCER	Agarwal R, 2003, NAT REV CANCER, V3, P502	37
Lu J, 2005, NATURE	Lu J, 2005, NATURE, V435, P834	37
Zhang S, 2008, CANCER RES	Zhang S, 2008, CANCER RES, V68, P4311	37
