# Supplementary material for: Therapy’s Shadow: A Short History of the Study of Resistance to Cancer Chemotherapy
Source: Front Pharmacol. 2013 May 7;4:58. doi: 10.3389/fphar.2013.00058 (PMC3646244; doi:10.3389/fphar.2013.00058)
Supplement: Supplementary file 1 [file 42120_Keating_Presentation1.PDF]

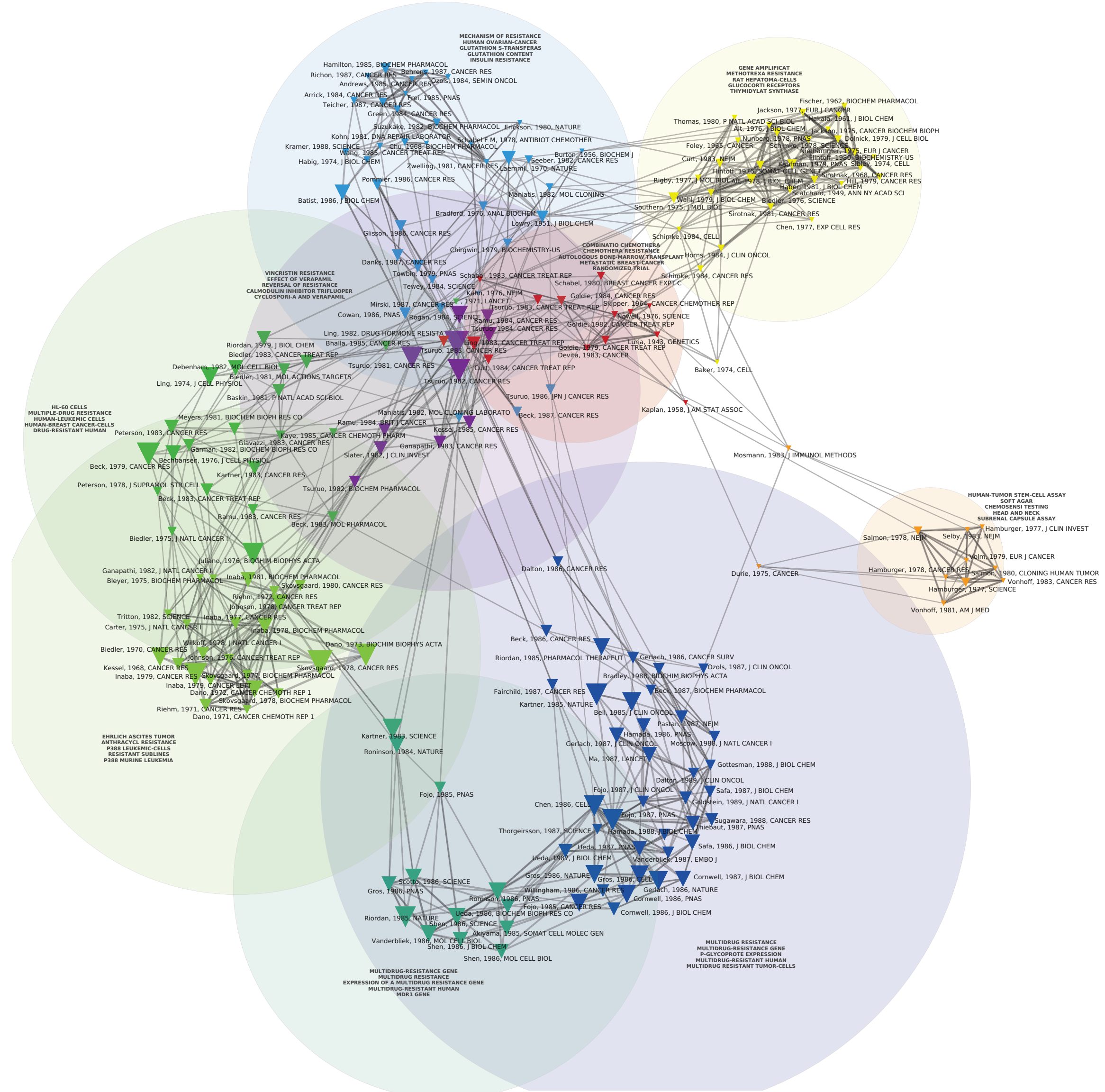
MECHANISM OF RESISTANCE
HUMAN OVARIAN-CANCER
GLUTATHION S-TRANSFERAS
GLUTATHION CONTENT
INSULIN RESISTANCE
Hamilton, 1985, BIOCHEM PHARMACOL
Behrens, 1987, CANCER RES
Richon, 1987, CANCER RES
Andrews, 1985, CANCER RES
Ozols, 1984, SEMIN ONCOL
Arrick, 1984, CANCER RES
Frei, 1985, PNAS
Teicher, 1987, CANCER RES
Green, 1984, CANCER RES
Suzukake, 1982, BIOCHEM PHARMACOL
Erickson, 1980, NATURE
Kohn, 1981, DNA REPAIR LABORATOR
Schabel F M, 1978, ANTIBIOT CHEMOTHER
Kramer, 1988, SCIENCE
Chu, 1968, BIOCHEM PHARMACOL
Wang, 1985, CANCER TREAT REP
Burton, 1956, BIOCHEM J
Habig, 1974, J BIOL CHEM
Seeber, 1982, CANCER RES
Zwelling, 1981, CANCER RES
Laemmli, 1970, NATURE
Pommier, 1986, CANCER RES
Maniatis, 1982, MOL CLONING
Batist, 1986, J BIOL CHEM
Bradford, 1976, ANAL BIOCHEM
Lowry, 1951, J BIOL CHEM
Glisson, 1986, CANCER RES
Chirgwin, 1979, BIOCHEMISTRY-US
Danks, 1987, CANCER RES
Towbin, 1979, PNAS
Tewey, 1984, SCIENCE
Mirski, 1987, CANCER RES
Cowan, 1986, PNAS
GENE AMPLIFICAT
METHOTREXA RESISTANCE
RAT HEPATOMA-CELLS
GLUCOCORTI RECEPTORS
THYMIDYLAT SYNTHASE
Fischer, 1962, BIOCHEM PHARMACOL
Jackson, 1977, EUR J CANCER
Thomas, 1980, P NATL ACAD SCI-BIOL
Hakala, 1961, J BIOL CHEM
Alt, 1976, J BIOL CHEM
Jackson, 1975, CANCER BIOCHEM BIOPH
Nunberg, 1978, PNAS
Dolnick, 1979, J CELL BIOL
Foley, 1965, CANCER
Schimke, 1978, SCIENCE
Niethammer, 1975, EUR J CANCER
Curt, 1983, NEJM
Flintoff, 1980, BIOCHEMISTRY-US
Kaufman, 1979, PNAS
Sibley, 1974, CELL
Flintoff, 1976, SOMAT CELL GENET
Sirotnak, 1968, CANCER RES
Rigby, 1977, J MOL BIOL
Alt, 1978, J BIOL CHEM
Hill, 1979, CANCER RES
Haber, 1981, J BIOL CHEM
Scatchard, 1949, ANN NY ACAD SCI
Wahl, 1979, J BIOL CHEM
Biedler, 1976, SCIENCE
Southern, 1975, J MOL BIOL
Sirotnak, 1981, CANCER RES
Chen, 1977, EXP CELL RES
Schimke, 1984, CELL
Horns, 1984, J CLIN ONCOL
Schimke, 1984, CANCER RES
VINCRISTIN RESISTANCE
EFFECT OF VERAPAMIL
REVERSAL OF RESISTANCE
CALMODULIN INHIBITOR TRIFLUOPER
CYCLOSPORI-A AND VERAPAMIL
COMBINATIO CHEMOTHERA
CHEMOTHERA RESISTANCE
AUTOLOGOUS BONE-MARROW TRANSPLANT
METASTATIC BREAST-CANCER
RANDOMIZED TRIAL
Schabel, 1983, CANCER TREAT REP
Schabel, 1980, BREAST CANCER EXPT C
Kahn, 1976, NEJM
Goldie, 1984, CANCER RES
1971, LANCET
Tsuruo, 1983, CANCER TREAT REP
Skipper, 1964, CANCER CHEMOTHER REP
Rogan, 1984, SCIENCE
Nowell, 1976, SCIENCE
Ramu, 1984, CANCER RES
Goldie, 1982, CANCER TREAT REP
Tsuruo, 1984, CANCER RES
Ling, 1982, DRUG HORMONE RESISTA
Luria, 1943, GENETICS
Bhalla, 1985, CANCER RES
Ling, 1983, CANCER TREAT REP
Goldie, 1979, CANCER TREAT REP
Riordan, 1979, J BIOL CHEM
Tsuruo, 1983, CANCER RES
Devita, 1983, CANCER
Biedler, 1983, CANCER TREAT REP
Tsuruo, 1981, CANCER RES
Curt, 1984, CANCER TREAT REP
Baker, 1974, CELL
Debenham, 1982, MOL CELL BIOL
Biedler, 1981, MOL ACTIONS TARGETS
Tsuruo, 1982, CANCER RES
Ling, 1974, J CELL PHYSIOL
Tsuruo, 1986, JPN J CANCER RES
Baskin, 1981, P NATL ACAD SCI-BIOL
Kaplan, 1958, J AM STAT ASSOC
Beck, 1987, CANCER RES
HL-60 CELLS
MULTIPLE-DRUG RESISTANCE
HUMAN-LEUKEMIC CELLS
HUMAN-BREAST CANCER-CELLS
DRUG-RESISTANT HUMAN
Maniatis, 1982, MOL CLONING LABORATO
Meyers, 1981, BIOCHEM BIOPH RES CO
Ramu, 1984, BRIT J CANCER
Kessel, 1985, CANCER RES
Peterson, 1983, CANCER RES
Kaye, 1985, CANCER CHEMOTH PHARM
Giavazzi, 1983, CANCER RES
Ganapathi, 1983, CANCER RES
Garman, 1982, BIOCHEM BIOPH RES CO
Mosmann, 1983, J IMMUNOL METHODS
Slater, 1982, J CLIN INVEST
Bech-hansen, 1976, J CELL PHYSIOL
Beck, 1979, CANCER RES
Kartner, 1983, CANCER RES
Peterson, 1978, J SUPRAMOL STR CELL
Tsuruo, 1982, BIOCHEM PHARMACOL
Beck, 1983, CANCER TREAT REP
HUMAN-TUMOR STEM-CELL ASSAY
SOFT AGAR
CHEMOSENSI TESTING
HEAD AND NECK
SUBRENAL CAPSULE ASSAY
Ramu, 1983, CANCER RES
Beck, 1983, MOL PHARMACOL
Hamburger, 1977, J CLIN INVEST
Salmon, 1978, NEJM
Selby, 1983, NEJM
Biedler, 1975, J NATL CANCER I
Volm, 1979, EUR J CANCER
Juliano, 1976, BIOCHIM BIOPHYS ACTA
Dalton, 1986, CANCER RES
Hamburger, 1978, CANCER RES
Salmon, 1980, CLONING HUMAN TUMOR
Ganapathi, 1982, J NATL CANCER I
Durie, 1975, CANCER
Vonhoff, 1983, CANCER RES
Inaba, 1981, BIOCHEM PHARMACOL
Hamburger, 1977, SCIENCE
Bleyer, 1975, BIOCHEM PHARMACOL
Skovsgaard, 1980, CANCER RES
Vonhoff, 1981, AM J MED
Riehm, 1972, CANCER RES
Johnson, 1978, CANCER TREAT REP
Tritton, 1982, SCIENCE
Inaba, 1977, CANCER RES
Carter, 1975, J NATL CANCER I
Inaba, 1978, BIOCHEM PHARMACOL
Beck, 1986, CANCER RES
Wilkoff, 1978, J NATL CANCER I
Dano, 1973, BIOCHIM BIOPHYS ACTA
Biedler, 1970, CANCER RES
Johnson, 1976, CANCER TREAT REP
Riordan, 1985, PHARMACOL THERAPEUT
Gerlach, 1986, CANCER SURV
Kessel, 1968, CANCER RES
Skovsgaard, 1978, CANCER RES
Ozols, 1987, J CLIN ONCOL
Inaba, 1979, CANCER RES
Skovsgaard, 1977, BIOCHEM PHARMACOL
Bradley, 1988, BIOCHIM BIOPHYS ACTA
Inaba, 1979, CANCER LETT
Dano, 1972, CANCER CHEMOTH REP 1
Fairchild, 1987, CANCER RES
Beck, 1987, BIOCHEM PHARMACOL
Skovsgaard, 1978, BIOCHEM PHARMACOL
Kartner, 1985, NATURE
Riehm, 1971, CANCER RES
Bell, 1985, J CLIN ONCOL
Dano, 1971, CANCER CHEMOTH REP 1
Pastan, 1987, NEJM
EHRLICH ASCITES TUMOR
ANTHRACYCL RESISTANCE
P388 LEUKEMIC-CELLS
RESISTANT SUBLINES
P388 MURINE LEUKEMIA
Kartner, 1983, SCIENCE
Hamada, 1986, PNAS
Gerlach, 1987, J CLIN ONCOL
Moscow, 1988, J NATL CANCER I
Roninson, 1984, NATURE
Ma, 1987, LANCET
Gottesman, 1988, J BIOL CHEM
Dalton, 1989, J CLIN ONCOL
Fojo, 1985, PNAS
Fojo, 1987, J CLIN ONCOL
Safa, 1987, J BIOL CHEM
Goldstein, 1989, J NATL CANCER I
Chen, 1986, CELL
Fojo, 1987, PNAS
Sugawara, 1988, CANCER RES
Thiebaut, 1987, PNAS
Thorgeirsson, 1987, SCIENCE
Hamada, 1988, J BIOL CHEM
Ueda, 1987, PNAS
Safa, 1986, J BIOL CHEM
Ueda, 1987, J BIOL CHEM
Vanderbliek, 1987, EMBO J
Gros, 1986, NATURE
Scotto, 1986, SCIENCE
Gros, 1986, CELL
Cornwell, 1987, J BIOL CHEM
Gros, 1986, PNAS
Willingham, 1986, CANCER RES
Gerlach, 1986, NATURE
Roninson, 1986, PNAS
Cornwell, 1986, PNAS
Fojo, 1985, CANCER RES
Ueda, 1986, BIOCHEM BIOPH RES CO
Cornwell, 1986, J BIOL CHEM
Riordan, 1985, NATURE
Shen, 1986, SCIENCE
Akiyama, 1985, SOMAT CELL MOLEC GEN
Vanderbliek, 1986, MOL CELL BIOL
Shen, 1986, J BIOL CHEM
Shen, 1986, MOL CELL BIOL
MULTIDRUG RESISTANCE
MULTIDRUG-RESISTANCE GENE
P-GLYCOPROTE EXPRESSION
MULTIDRUG-RESISTANT HUMAN
MULTIDRUG RESISTANT TUMOR-CELLS
MULTIDRUG-RESISTANCE GENE
MULTIDRUG RESISTANCE
EXPRESSION OF A MULTIDRUG RESISTANCE GENE
MULTIDRUG-RESISTANT HUMAN
MDR1 GENE
