# Supplementary material for: Therapy’s Shadow: A Short History of the Study of Resistance to Cancer Chemotherapy
Source: Front Pharmacol. 2013 May 7;4:58. doi: 10.3389/fphar.2013.00058 (PMC3646244; doi:10.3389/fphar.2013.00058)
Supplement: Supplementary file 2 [file 42120_Keating_Presentation2.PDF]

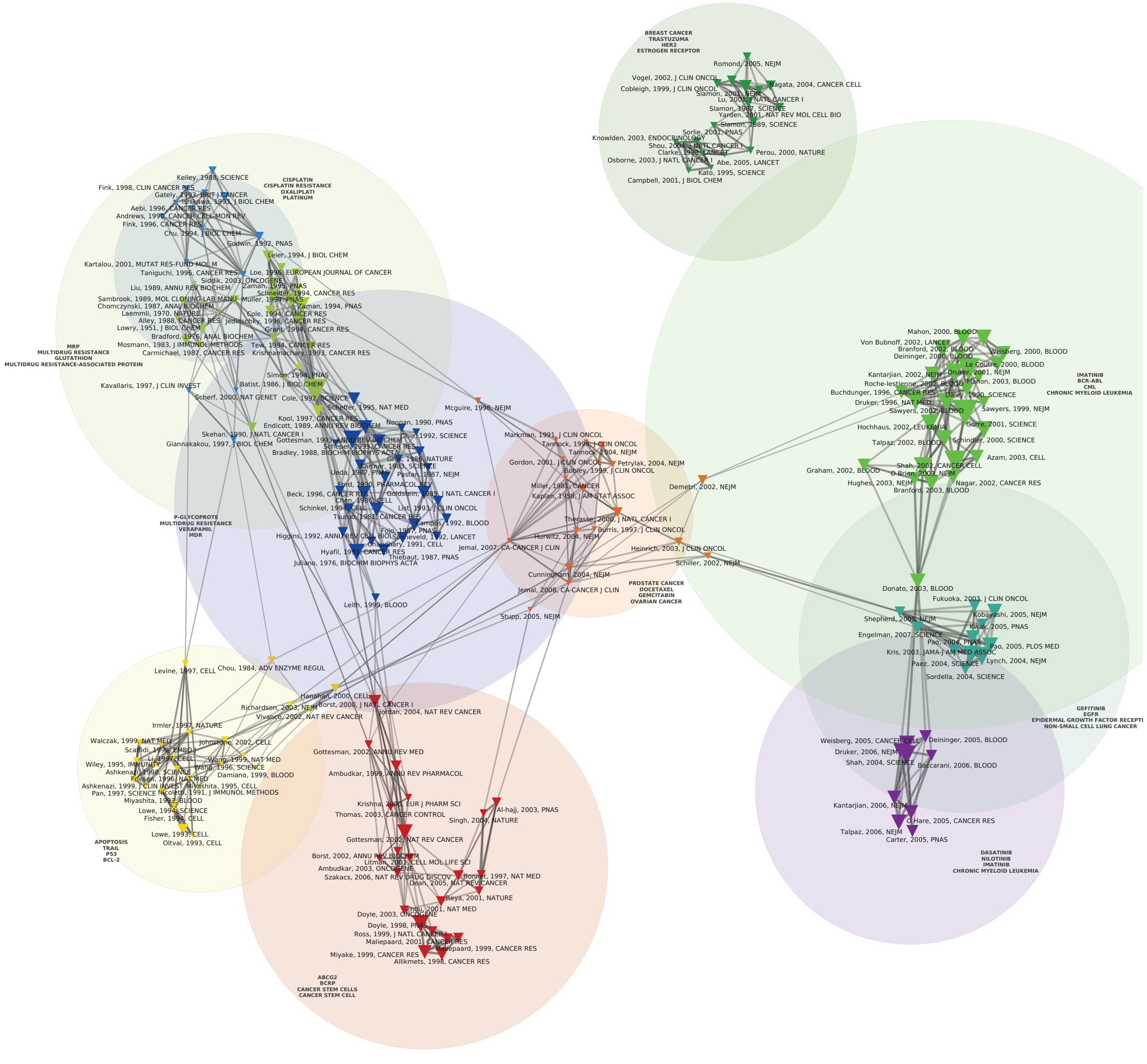
BREAST CANCER
TRASTUZUMA
HER2
ESTROGEN RECEPTOR
Romond, 2005, NEJM
Vogel, 2002, J CLIN ONCOL
Nagata, 2004, CANCER CELL
Cobleigh, 1999, J CLIN ONCOL
Slamon, 2001, NEJM
Lu, 2001, J NATL CANCER I
Slamon, 1987, SCIENCE
Yarden, 2001, NAT REV MOL CELL BIO
Slamon, 1989, SCIENCE
Sorlie, 2001, PNAS
Knowlden, 2003, ENDOCRINOLOGY
Shou, 2004, J NATL CANCER I
Clarke, 1998, LANCET
Perou, 2000, NATURE
Osborne, 2003, J NATL CANCER I
Abe, 2005, LANCET
Kato, 1995, SCIENCE
Campbell, 2001, J BIOL CHEM
Kelley, 1988, SCIENCE
Fink, 1998, CLIN CANCER RES
Gately, 1993, BRIT J CANCER
Ishikawa, 1993, J BIOL CHEM
Aebi, 1996, CANCER RES
Andrews, 1990, CANCER CELL-MON REV
Fink, 1996, CANCER RES
Chu, 1994, J BIOL CHEM
Godwin, 1992, PNAS
CISPLATIN
CISPLATIN RESISTANCE
OXALIPLATI
PLATINUM
Leier, 1994, J BIOL CHEM
Kartalou, 2001, MUTAT RES-FUND MOL M
Taniguchi, 1996, CANCER RES
Loe, 1996, EUROPEAN JOURNAL OF CANCER
Siddik, 2003, ONCOGENE
Liu, 1989, ANNU REV BIOCHEM
Zaman, 1995, PNAS
Schneider, 1994, CANCER RES
Sambrook, 1989, MOL CLONING LAB MANU
Muller, 1994, PNAS
Chomczynski, 1987, ANAL BIOCHEM
Zaman, 1994, PNAS
Laemmli, 1970, NATURE
Cole, 1994, CANCER RES
Alley, 1988, CANCER RES
Jedlitschky, 1996, CANCER RES
Lowry, 1951, J BIOL CHEM
Grant, 1994, CANCER RES
Bradford, 1976, ANAL BIOCHEM
Mosmann, 1983, J IMMUNOL METHODS
Tew, 1994, CANCER RES
Carmichael, 1987, CANCER RES
Krishnamachary, 1993, CANCER RES
MRP
MULTIDRUG RESISTANCE
GLUTATHION
MULTIDRUG RESISTANCE-ASSOCIATED PROTEIN
Simon, 1994, PNAS
Kavallaris, 1997, J CLIN INVEST
Batist, 1986, J BIOL CHEM
Scherf, 2000, NAT GENET
Cole, 1992, SCIENCE
Scheffer, 1995, NAT MED
Mcguire, 1996, NEJM
Kool, 1997, CANCER RES
Endicott, 1989, ANNU REV BIOCHEM
Noonan, 1990, PNAS
Skehan, 1990, J NATL CANCER I
Gottesman, 1993, ANNU REV BIOCHEM
Chin, 1992, SCIENCE
Giannakakou, 1997, J BIOL CHEM
Scheper, 1993, CANCER RES
Bradley, 1988, BIOCHIM BIOPHYS ACTA
Gros, 1986, NATURE
Kartner, 1983, SCIENCE
Ueda, 1987, PNAS
Pastan, 1987, NEJM
Ford, 1990, PHARMACOL REV
Beck, 1996, CANCER RES
Goldstein, 1989, J NATL CANCER I
Chen, 1986, CELL
Schinkel, 1994, CELL
List, 1993, J CLIN ONCOL
Tsuruo, 1981, CANCER RES
Campos, 1992, BLOOD
Fojo, 1987, PNAS
Higgins, 1992, ANNU REV CELL BIOL
Sonneveld, 1992, LANCET
Chaudhary, 1991, CELL
Hyafil, 1993, CANCER RES
Thiebaut, 1987, PNAS
Juliano, 1976, BIOCHIM BIOPHYS ACTA
Leith, 1999, BLOOD
P-GLYCOPROTE
MULTIDRUG RESISTANCE
VERAPAMIL
MDR
Markman, 1991, J CLIN ONCOL
Tannock, 1996, J CLIN ONCOL
Tannock, 2004, NEJM
Gordon, 2001, J CLIN ONCOL
Petrylak, 2004, NEJM
Bubley, 1999, J CLIN ONCOL
Miller, 1981, CANCER
Demetri, 2002, NEJM
Kaplan, 1958, J AM STAT ASSOC
Therasse, 2000, J NATL CANCER I
Burris, 1997, J CLIN ONCOL
Hurwitz, 2004, NEJM
Jemal, 2007, CA-CANCER J CLIN
Heinrich, 2003, J CLIN ONCOL
Schiller, 2002, NEJM
Cunningham, 2004, NEJM
Jemal, 2008, CA-CANCER J CLIN
PROSTATE CANCER
DOCETAXEL
GEMCITABIN
OVARIAN CANCER
Stupp, 2005, NEJM
Mahon, 2000, BLOOD
Von Bubnoff, 2002, LANCET
Branford, 2002, BLOOD
Weisberg, 2000, BLOOD
Deininger, 2000, BLOOD
Le Coutre, 2000, BLOOD
Kantarjian, 2002, NEJM
Druker, 2001, NEJM
IMATINIB
BCR-ABL
CML
CHRONIC MYELOID LEUKEMIA
Roche-lestienne, 2002, BLOOD
Mahon, 2003, BLOOD
Buchdunger, 1996, CANCER RES
Daley, 1990, SCIENCE
Druker, 1996, NAT MED
Sawyers, 1999, NEJM
Sawyers, 2002, BLOOD
Gorre, 2001, SCIENCE
Hochhaus, 2002, LEUKEMIA
Talpaz, 2002, BLOOD
Schindler, 2000, SCIENCE
Azam, 2003, CELL
Shah, 2002, CANCER CELL
Graham, 2002, BLOOD
O Brien, 2003, NEJM
Hughes, 2003, NEJM
Nagar, 2002, CANCER RES
Branford, 2003, BLOOD
Donato, 2003, BLOOD
Fukuoka, 2003, J CLIN ONCOL
Shepherd, 2005, NEJM
Kobayashi, 2005, NEJM
Kwak, 2005, PNAS
Engelman, 2007, SCIENCE
Pao, 2004, PNAS
Pao, 2005, PLOS MED
Kris, 2003, JAMA-J AM MED ASSOC
Lynch, 2004, NEJM
Paez, 2004, SCIENCE
Sordella, 2004, SCIENCE
GEFITINIB
EGFR
EPIDERMAL GROWTH FACTOR RECEPTO
NON-SMALL CELL LUNG CANCER
Weisberg, 2005, CANCER CELL
Deininger, 2005, BLOOD
Druker, 2006, NEJM
Shah, 2004, SCIENCE
Baccarani, 2006, BLOOD
Kantarjian, 2006, NEJM
O Hare, 2005, CANCER RES
Talpaz, 2006, NEJM
Carter, 2005, PNAS
DASATINIB
NILOTINIB
IMATINIB
CHRONIC MYELOID LEUKEMIA
Levine, 1997, CELL
Chou, 1984, ADV ENZYME REGUL
Hanahan, 2000, CELL
Richardson, 2003, NEJM
Borst, 2000, J NATL CANCER I
Jordan, 2004, NAT REV CANCER
Vivanco, 2002, NAT REV CANCER
Irmler, 1997, NATURE
Walczak, 1999, NAT MED
Johnstone, 2002, CELL
Scaffidi, 1998, EMBO J
Li, 1997, CELL
Wang, 1999, NAT MED
Wiley, 1995, IMMUNITY
Wang, 1996, SCIENCE
Ashkenazi, 1998, SCIENCE
Damiano, 1999, BLOOD
Friesen, 1996, NAT MED
Ashkenazi, 1999, J CLIN INVEST
Miyashita, 1995, CELL
Pan, 1997, SCIENCE
Nicoletti, 1991, J IMMUNOL METHODS
Miyashita, 1994, BLOOD
Lowe, 1994, SCIENCE
Fisher, 1994, CELL
Lowe, 1993, CELL
Oltvai, 1993, CELL
APOPTOSIS
TRAIL
P53
BCL-2
Gottesman, 2002, ANNU REV MED
Ambudkar, 1999, ANNU REV PHARMACOL
Krishna, 2000, EUR J PHARM SCI
Al-hajj, 2003, PNAS
Thomas, 2003, CANCER CONTROL
Singh, 2004, NATURE
Gottesman, 2002, NAT REV CANCER
Borst, 2002, ANNU REV BIOCHEM
Litman, 2001, CELL MOL LIFE SCI
Ambudkar, 2003, ONCOGENE
Szakacs, 2006, NAT REV DRUG DISCOV
Bonnet, 1997, NAT MED
Dean, 2005, NAT REV CANCER
Reya, 2001, NATURE
Zhou, 2001, NAT MED
Doyle, 2003, ONCOGENE
Doyle, 1998, PNAS
Ross, 1999, J NATL CANCER I
Maliepaard, 2001, CANCER RES
Maliepaard, 1999, CANCER RES
Miyake, 1999, CANCER RES
Allikmets, 1998, CANCER RES
ABCG2
BCRP
CANCER STEM CELLS
CANCER STEM CELL
