# Supplementary material for: Therapy’s Shadow: A Short History of the Study of Resistance to Cancer Chemotherapy
Source: Front Pharmacol. 2013 May 7;4:58. doi: 10.3389/fphar.2013.00058 (PMC3646244; doi:10.3389/fphar.2013.00058)
Supplement: Supplementary file 3 [file 42120_Keating_Presentation3.PDF]

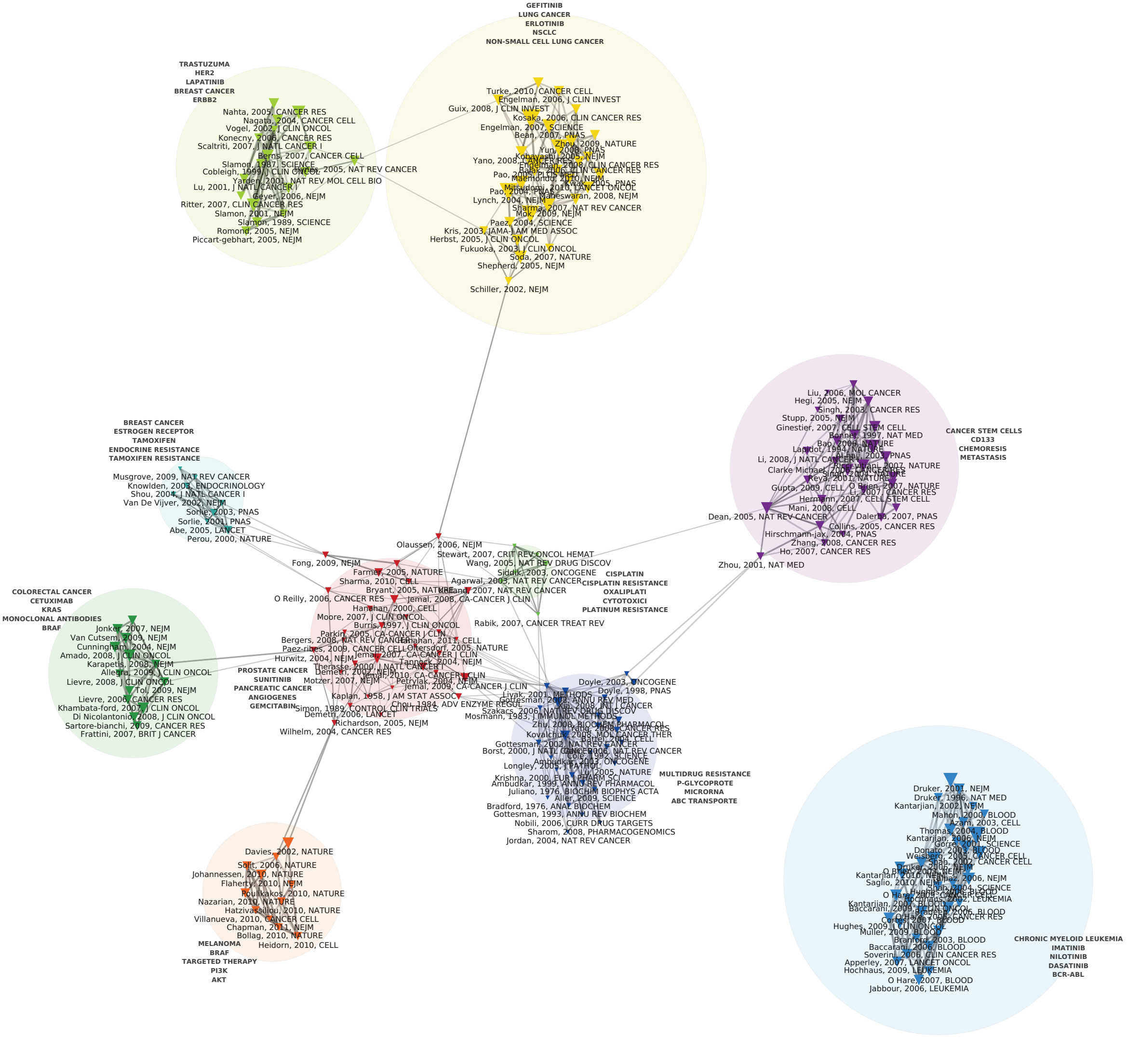
GEFITINIB
LUNG CANCER
ERLOTINIB
NSCLC
NON-SMALL CELL LUNG CANCER
Turke, 2010, CANCER CELL
Engelman, 2006, J CLIN INVEST
Guix, 2008, J CLIN INVEST
Kosaka, 2006, CLIN CANCER RES
Engelman, 2007, SCIENCE
Bean, 2007, PNAS
Zhou, 2009, NATURE
Yun, 2008, PNAS
Kobayashi, 2005, NEJM
Yano, 2008, CANCER RES
Maheswaran, 2008, NEJM
Lynch, 2004, NEJM
Sharma, 2007, NAT REV CANCER
Mok, 2009, NEJM
Paez, 2004, SCIENCE
Kris, 2003, JAMA-J AM MED ASSOC
Herbst, 2005, J CLIN ONCOL
Fukuoka, 2003, J CLIN ONCOL
Soda, 2007, NATURE
Shepherd, 2005, NEJM
Schiller, 2002, NEJM
TRASTUZUMA
HER2
LAPATINIB
BREAST CANCER
ERBB2
Nahta, 2005, CANCER RES
Nagata, 2004, CANCER CELL
Vogel, 2002, J CLIN ONCOL
Konecny, 2006, CANCER RES
Scaltriti, 2007, J NATL CANCER I
Berns, 2007, CANCER CELL
Slamon, 1987, SCIENCE
Cobleigh, 1999, J CLIN ONCOL
Yarden, 2001, NAT REV MOL CELL BIO
Lu, 2001, J NATL CANCER I
Geyer, 2006, NEJM
Ritter, 2007, CLIN CANCER RES
Slamon, 2001, NEJM
Slamon, 1989, SCIENCE
Romond, 2005, NEJM
Piccart-gebhart, 2005, NEJM
Liu, 2006, MOL CANCER
Hegi, 2005, NEJM
Singh, 2003, CANCER RES
Stupp, 2005, NEJM
Ginestier, 2007, CELL STEM CELL
Bonnet, 1997, NAT MED
Bao, 2006, NATURE
Lapidot, 1994, NATURE
Al-hajj, 2003, PNAS
Li, 2008, J NATL CANCER I
Clarke Michael, 2006, CANCER RES
Reya, 2001, NATURE
Gupta, 2009, CELL
O Brien, 2007, NATURE
Li, 2007, CANCER RES
Hermann, 2007, CELL STEM CELL
Mani, 2008, CELL
Dean, 2005, NAT REV CANCER
Dalerba, 2007, PNAS
Collins, 2005, CANCER RES
Hirschmann-jax, 2004, PNAS
Zhang, 2008, CANCER RES
Ho, 2007, CANCER RES
Zhou, 2001, NAT MED
CANCER STEM CELLS
CD133
CHEMORESIS
METASTASIS
BREAST CANCER
ESTROGEN RECEPTOR
TAMOXIFEN
ENDOCRINE RESISTANCE
TAMOXIFEN RESISTANCE
Musgrove, 2009, NAT REV CANCER
Knowlden, 2003, ENDOCRINOLOGY
Shou, 2004, J NATL CANCER I
Van De Vijver, 2002, NEJM
Sorlie, 2003, PNAS
Sorlie, 2001, PNAS
Abe, 2005, LANCET
Perou, 2000, NATURE
Olaussen, 2006, NEJM
Fong, 2009, NEJM
Stewart, 2007, CRIT REV ONCOL HEMAT
Wang, 2005, NAT REV DRUG DISCOV
Siddik, 2003, ONCOGENE
Agarwal, 2003, NAT REV CANCER
Kelland, 2007, NAT REV CANCER
Rabik, 2007, CANCER TREAT REV
CISPLATIN
CISPLATIN RESISTANCE
OXALIPLATI
CYTOTOXICI
PLATINUM RESISTANCE
Farmer, 2005, NATURE
Sharma, 2010, CELL
Bryant, 2005, NATURE
O Reilly, 2006, CANCER RES
Jemal, 2008, CA-CANCER J CLIN
Hanahan, 2000, CELL
Moore, 2007, J CLIN ONCOL
Burris, 1997, J CLIN ONCOL
Parkin, 2005, CA-CANCER J CLIN
Bergers, 2008, NAT REV CANCER
Hanahan, 2011, CELL
Paez-ribes, 2009, CANCER CELL
Oltersdorf, 2005, NATURE
Hurwitz, 2004, NEJM
Jemal, 2007, CA-CANCER J CLIN
Tannock, 2004, NEJM
Therasse, 2000, J NATL CANCER I
Demetri, 2002, NEJM
Jemal, 2010, CA-CANCER J CLIN
Motzer, 2007, NEJM
Petrylak, 2004, NEJM
Jemal, 2009, CA-CANCER J CLIN
Kaplan, 1958, J AM STAT ASSOC
Chou, 1984, ADV ENZYME REGUL
Simon, 1989, CONTROL CLIN TRIALS
Demetri, 2006, LANCET
Richardson, 2005, NEJM
Wilhelm, 2004, CANCER RES
PROSTATE CANCER
SUNITINIB
PANCREATIC CANCER
ANGIOGENES
GEMCITABIN
COLORECTAL CANCER
CETUXIMAB
KRAS
MONOCLONAL ANTIBODIES
BRAF
Jonker, 2007, NEJM
Van Cutsem, 2009, NEJM
Cunningham, 2004, NEJM
Amado, 2008, J CLIN ONCOL
Karapetis, 2008, NEJM
Allegra, 2009, J CLIN ONCOL
Lievre, 2008, J CLIN ONCOL
Tol, 2009, NEJM
Lievre, 2006, CANCER RES
Khambata-ford, 2007, J CLIN ONCOL
Di Nicolantonio, 2008, J CLIN ONCOL
Sartore-bianchi, 2009, CANCER RES
Frattini, 2007, BRIT J CANCER
Doyle, 2003, ONCOGENE
Doyle, 1998, PNAS
Liyak, 2001, METHODS
Gottesman, 2002, ANNU REV MED
Szakacs, 2006, NAT REV DRUG DISCOV
Mosmann, 1983, J IMMUNOL METHODS
Zhu, 2008, BIOCHEM PHARMACOL
Gottesman, 2002, NAT REV CANCER
Borst, 2000, J NATL CANCER I
Cole, 1992, SCIENCE
Ambudkar, 2003, ONCOGENE
Longley, 2005, J PATHOL
Krishna, 2000, EUR J PHARM SCI
Ambudkar, 1999, ANNU REV PHARMACOL
Juliano, 1976, BIOCHIM BIOPHYS ACTA
Aller, 2009, SCIENCE
Bradford, 1976, ANAL BIOCHEM
Gottesman, 1993, ANNU REV BIOCHEM
Nobili, 2006, CURR DRUG TARGETS
Sharom, 2008, PHARMACOGENOMICS
Jordan, 2004, NAT REV CANCER
MULTIDRUG RESISTANCE
P-GLYCOPROTE
MICRORNA
ABC TRANSPORTE
Davies, 2002, NATURE
Solit, 2006, NATURE
Johannessen, 2010, NATURE
Flaherty, 2010, NEJM
Poulikakos, 2010, NATURE
Nazarian, 2010, NATURE
Hatzivassiliou, 2010, NATURE
Villanueva, 2010, CANCER CELL
Chapman, 2011, NEJM
Bollag, 2010, NATURE
Heidorn, 2010, CELL
MELANOMA
BRAF
TARGETED THERAPY
PI3K
AKT
Druker, 2001, NEJM
Druker, 1996, NAT MED
Kantarjian, 2002, NEJM
Mahon, 2000, BLOOD
Azam, 2003, CELL
Thomas, 2004, BLOOD
Kantarjian, 2006, NEJM
Gorre, 2001, SCIENCE
Donato, 2003, BLOOD
Weisberg, 2005, CANCER CELL
Shah, 2002, CANCER CELL
Kantarjian, 2010, NEJM
Talpaz, 2006, NEJM
Saglio, 2010, NEJM
Shah, 2004, SCIENCE
Kantarjian, 2007, BLOOD
Baccarani, 2009, J CLIN ONCOL
Branford, 2006, BLOOD
Cortes, 2007, BLOOD
Hughes, 2009, J CLIN ONCOL
Muller, 2009, BLOOD
Branford, 2003, BLOOD
Baccarani, 2006, BLOOD
Soverini, 2006, CLIN CANCER RES
Apperley, 2007, LANCET ONCOL
Hochhaus, 2009, LEUKEMIA
O Hare, 2007, BLOOD
Jabbour, 2006, LEUKEMIA
CHRONIC MYELOID LEUKEMIA
IMATINIB
NILOTINIB
DASATINIB
BCR-ABL
